# Gut mycobiota dysbiosis and an emergent state of “co-dysbiosis” are associated with IgE sensitization in children with comorbid allergic rhinitis and constipation

**DOI:** 10.3389/fimmu.2025.1745580

**Published:** 2026-01-23

**Authors:** Haiying Liu, Liqing Liang, Chunyan Wang, Rongrong Luo, Qiuhua Luo, Congfu Huang

**Affiliations:** 1 Department of Pediatrics, Shenzhen Maternity and Child Healthcare Hospital, Southern Medical University, Shenzhen, China; 2 Department of Pediatrics, Longgang District Maternity & Child Healthcare Hospital of Shenzhen City (Longgang Maternity and Child Institute of Shantou University Medical College), Shenzhen, China; 3 Department of Pediatrics, Shenzhen Fourth People’s Hospital (Sami Medical Center), Shenzhen, China

**Keywords:** allergic rhinitis, children, co-dysbiosis, cross-kingdom network, functional constipation, fungal dysbiosis, gut mycobiota, IgE

## Abstract

**Background:**

The comorbidity of allergic rhinitis (AR) and functional constipation (FC), termed ARFC, implies shared gut–immune pathways. Although bacterial dysbiosis has been implicated, the role of the gut mycobiota (fungal community) in this specific comorbidity remains unexplored.

**Methods:**

This pilot case-control study characterized the gut mycobiota in 19 ARFC and 17 healthy control (HC) children aged 3–6 years using metagenomic sequencing. Fungal community structure, taxonomic composition, and correlations with IgE levels were analyzed. Cross-kingdom bacterial–fungal interaction networks were constructed, and functional potential was predicted.

**Results:**

Alpha diversity was comparable, whereas beta diversity revealed significant structural shifts in the ARFC gut mycobiota. Key immunomodulatory fungi, including Cenococcum, Dentiscutata, Ambispora, and Saccharomyces, were markedly depleted in ARFC. These taxa served as top discriminators in random forest models and exhibited significant inverse correlations with total and allergen-specific IgE levels. Cross-kingdom network analysis identified dramatic ecological restructuring: the HC network was characterized by prevalent competitive interactions, whereas the ARFC network shifted exclusively to positive correlations, a state termed “co-dysbiosis.” No significant differences were observed in predicted KEGG functional pathways.

**Conclusion:**

This study provides the first evidence that gut mycobiota dysbiosis—marked by depletion of immunoregulatory fungi and an ecological shift toward cooperative interkingdom interactions (“co-dysbiosis”)—is associated with IgE sensitization in ARFC children. These findings position the gut mycobiota as a novel element of the gut–nose axis in allergic disease, warranting further investigation.

## Graphical Abstract

**Figure:**
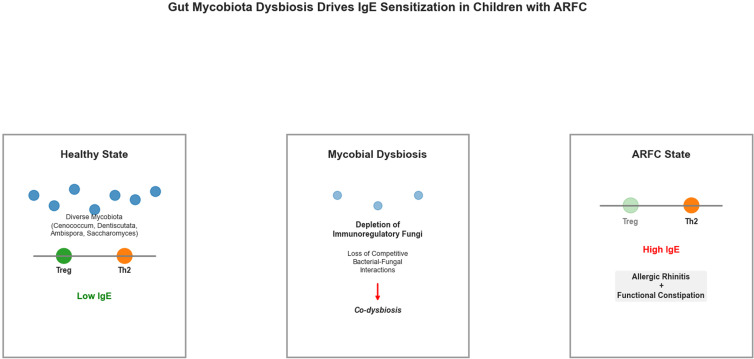

Proposed mechanism linking gut mycobiota dysbiosis to IgE sensitization in children with comorbid allergic rhinitis and functional constipation (ARFC).

This model hypothesizes how gut mycobiota dysbiosis may contribute to IgE sensitization in ARFC. In the healthy state (left), a diverse mycobiota, including genera such as Cenococcum, Dentiscutata, Ambispora, and Saccharomyces, may support immune homeostasis by promoting regulatory T cell (Treg) responses and suppressing IgE production. In ARFC (right), depletion of these fungi is proposed to disrupt immunoregulatory pathways (e.g., Dectin-1 signaling, SCFA production), leading to a predominant Th2 response, elevated IgE, and clinical manifestations.

## 1 Introduction

Allergic rhinitis (AR) ranks among the most prevalent chronic inflammatory diseases in pediatric populations, with a steadily increasing global prevalence that significantly impairs quality of life and disrupts sleep (1, 2). Clinically, AR is frequently comorbid with functional constipation (FC)—a gastrointestinal disorder characterized by difficult and infrequent bowel movements—due to notable epidemiological links (3, 4). Evidence from population-based cohorts suggests constipation may be a risk factor for subsequent AR development (3), potentially exacerbating allergic symptoms (5). This clinical synergy indicates shared pathophysiology mediated through the gut–microbiota–host immune axis, as evidenced by bacterial dysbiosis in both conditions (6–8). However, the high impact of ARFC comorbidity on pediatric quality of life contrasts with scarce research on its shared mechanisms. Current management often addresses each condition in isolation, highlighting an unmet need for integrated approaches.

Dysbiosis of the bacterial microbiota is well-documented in isolated AR and constipation, implicating roles in immune regulation, barrier integrity, and immunomodulatory metabolite production (e.g., short-chain fatty acids) (6–10). In contrast, the gut mycobiota—the fungal component—remains understudied (11, 12). Recent research illuminates fungi as active immunomodulators via Dectin-1 and CARD9 signaling (13–15), often through interactions with bacteria (16) and host immune cells (e.g., CX3CR1+ mononuclear phagocytes) (17). Genomic resources highlight fungal diversity and disease associations (18), yet their role in ARFC remains unknown.

Fungi engage host pattern-recognition receptors (e.g., Dectin-1, TLRs), initiating cascades such as the SYK–CARD9 axis, which regulates antifungal immunity and allergic inflammation (13, 14, 19). The “gut–fungi–lung axis” demonstrates intestinal fungal influence on remote mucosal sites via systemic immune activation (20, 21), with evidence supporting gut-lung crosstalk in asthma (22). For instance, gut-resident CX3CR1+ phagocytes respond to fungal dysbiosis, aggravating allergic airway disease (20). Similarly, expansion of Wallemia mellicola enhances allergic airway severity in mice (23). Building on the “gut–fungi–lung axis” (20, 21, 24) and gut-nose communication in allergy (25–27), a “gut–fungi–nose axis” is hypothesized to underlie ARFC comorbidity, supported by fungal associations with immune-mediated conditions (e.g., asthma, inflammatory bowel disease) (14, 19, 28).

Significant knowledge gaps persist. First, while fungi have been examined in isolated AR or FC (19, 29), the gut mycobiota in ARFC children remains uncharacterized. Second, the potential for fungal dysbiosis to disrupt immune homeostasis—via mechanisms like barrier impairment and SYK–CARD9 activation (30, 31)—is unexplored in this context.

Therefore, this pilot study characterized the gut mycobiota in ARFC children versus healthy controls (HC) using metagenomic sequencing, testing the hypothesis that its composition correlates with IgE-mediated sensitization. To our knowledge, this is the first mycobiota profiling in ARFC comorbidity, aiming to generate hypotheses about the gut–fungi–nose axis.

## 2 Materials and methods

### 2.1 Study participants and sample collection

This case-control study was conducted in the Department of Pediatrics at Longgang District Maternity & Child Healthcare Hospital of Shenzhen between June 2024 and June 2025. The study protocol was approved by the Institutional Ethics Committee (Approval No: KYXMLL-01-CZGC-14-2-1), and written informed consent was obtained from guardians. Thirty-six children aged 3–6 years were enrolled (19 ARFC, 17 HC). FC diagnosis followed Rome IV criteria (32), including symptoms persisting ≥1 month. AR diagnosis adhered to Chinese Guidelines (2022) (33). Demographic characteristics are summarized in **Table 1**. No significant differences in age (unpaired t-test) or gender distribution (χ² test) were observed (p > 0.05). HC children had no history of allergic diseases, infections, or antibiotic use within one month prior. Exclusion criteria included chronic comorbidities and recent probiotic/immunomodulator use. Fecal samples were collected and stored at −80°C.

**Table 1:** Demographic and clinical characteristics of children with allergic rhinitis and functional constipation (ARFC) and healthy controls (HC).

Characteristic^a^	ARFC (n = 19)	HC (n = 17)	*p*-value
Age (years)^a^	4.8 ± 0.9	4.5 ± 1.1	0.351
Gender (Male/Female)^b^	10/9	9/8	0.942
Height (cm)^a^	105.3 ± 6.2	107.1 ± 5.8	0.324
Weight (kg)^a^	18.2 ± 2.4	17.8 ± 2.1	0.568
Allergy symptom score^a^	8.5 ± 2.1	1.2 ± 0.8	<0.001
Constipation prevalence, n (%)^b^	19 (100%)	0 (0%)	<0.001
Total IgE (IU/mL)^a^	285.6 ± 102.4	65.3 ± 28.7	<0.001

1. Data are presented as mean ± standard deviation or number (percentage); 2.a Continuous variables were compared using the unpaired t-test; 3.b Categorical variables were compared using the Chi-square test; 4. Allergy symptom score was assessed based on standardized pediatric allergic rhinitis diagnostic criteria.

### 2.2 Sample handling and storage

Fecal samples were collected using sterile swabs, flash-frozen in liquid nitrogen, and stored at −80°C within 30 minutes. Transportation occurred on dry ice within 2 hours, with processing completed within 24 hours. Samples were lyophilized and homogenized prior to DNA extraction.

### 2.3 Serum IgE measurement

Venous blood was collected at enrollment. Serum was separated by centrifugation (3000 rpm, 10 min) and stored at −80°C.

Total IgE: Quantified via Human IgE ELISA Kit (Elabscience, Catalog No. E-EL-H0104c) per manufacturer instructions. Duplicate assays expressed IgE in IU/mL (detection limit: 0.1 IU/mL; CV < 8%).

Allergen-specific IgE: Quantified against pediatric allergens (e.g., dust mites, shrimp) via AllergyScreen^®^ microarray (Mediwiss Analytic GmbH), based on immunoCAP^®^ FEIA. Sensitization threshold: ≥0.35 kU_a_/L. Manufacturer controls were included.

### 2.4 Metagenomic sequencing and fungal community analysis

#### 2.4.1 DNA extraction and quality control

Total genomic DNA was extracted from 0.2 g stool using FastPure Stool DNA Isolation Kit (MJYH). Concentration and purity were assessed via Synergy HTX reader (BioTek) and NanoDrop 2000 (Thermo Fisher). Samples with DNA ≥ 20 ng/μL and A260/A280 1.8–2.0 were retained; integrity was confirmed by 1% agarose gel electrophoresis.

#### 2.4.2 Library preparation and sequencing

Qualified DNA (100 ng) was fragmented to ~350 bp via Covaris M220 ultrasonicator. Paired-end libraries were prepared with NEXTFLEX Rapid DNA-Seq Kit (Bioo Scientific). Shotgun sequencing was performed on Illumina NovaSeq X Plus (Majorbio Bio-Pharm Technology Co.), yielding ≥10 Gb/sample.

#### 2.4.3 Sequence preprocessing and fungal contig identification

Raw data were processed on Majorbio Cloud Platform. Adapters and low-quality reads were removed using fastp (v0.23.0). Human reads (GRCh38) were excluded via BWA (v0.7.17). High-quality reads were assembled *de novo* with MEGAHIT (v1.1.2), retaining contigs ≥ 300 bp.

Fungal contigs were classified using VirSorter2 (v2.2.4; categories 1,2,4,5) or DeepVirFinder (v1.0; score >0.9, p<0.05). Contig quality was assessed with CheckV (v1.0.1; completeness ≥50%, contamination ≤10%). Host/bacterial sequences were removed via Bowtie2 (v2.4.5) against RefSeq genomes. Statistics are in **Supplementary Table S1**.

#### 2.4.4 Taxonomic annotation and abundance profiling

Open reading frames (ORFs) were predicted via Prodigal (v2.6.3), retaining ≥100-bp sequences. A non-redundant gene catalog was constructed with CD-HIT (v4.6.1; 90% identity/coverage). Gene abundance was estimated by mapping reads to the catalog via SOAPaligner (v2.21; 95% identity). Taxonomic annotation aligned genes against NCBI NR fungi database using DIAMOND (v2.0.13; e-value ≤1e−5). Dietary contigs were excluded.

#### 2.4.5 Functional annotation

Functional potential was assessed by aligning the gene catalog against KEGG Orthology (KO) via DIAMOND (e-value ≤1e−5). Abundances were summarized at Level 3 pathways.

### 2.5 Statistical analysis

All statistical and bioinformatic analyses were conducted in R (version 4.3.1). Alpha diversity indices (Chao1 and Shannon) were compared between groups using the Wilcoxon rank-sum test. Beta diversity was assessed based on Bray–Curtis distances and visualized via principal coordinate analysis (PCoA). Permutational multivariate analysis of variance (PERMANOVA) with 9999 permutations was used to examine group differences in fungal community structure, and Procrustes analysis was applied to assess overall configuration similarity.

Differential abundance of fungal taxa across taxonomic levels (phylum, family, genus) was assessed using linear discriminant analysis effect size (LEfSe). A threshold of LDA score > 2 and FDR-adjusted p-value < 0.05 was applied to define statistically significant taxa.

A random forest model implemented via the randomForest package was used to identify key fungal genera and clinical variables discriminating between ARFC and HC participants. The model was trained using 10-fold cross-validation, and variable importance was evaluated based on the MeanDecreaseGini metric. Model performance was assessed using the area under the receiver operating characteristic curve (AUC) and out-of-bag (OOB) error estimation.

Associations between fungal genera and clinical variables (total IgE and allergen-specific IgE levels) were examined using Spearman’s rank correlation. To assess the functional potential of the gut mycobiota, quality-filtered reads were aligned against the KEGG Orthology (KO) database using Diamond (v2.1.8) with an e-value threshold of 1e−5. KO abundances were summarized at Level 3 pathway categories. Differential abundance of KEGG pathways between groups was assessed using the ReporterScore algorithm. Pathways with an absolute ReporterScore > |1.5| and a corrected significance designation (“yes”) were considered significantly enriched. Pathways labeled “ns” (not significant) were retained for exploratory interpretation.

### 2.6 Bacterial–fungal interaction network analysis

To elucidate the ecological relationships between gut bacteria and fungi, cross-kingdom interaction networks were constructed separately for the ARFC and HC groups. To ensure maximum comparability and integration, bacterial abundance profiles were directly derived from the identical whole-genome shotgun metagenomic sequencing dataset used for mycobiota characterization. This approach guarantees that all downstream correlations are inferred from the same biological sample aliquots and sequencing runs, providing a unified basis for interkingdom network analysis.

Network Construction: For each group, pairwise Spearman’s rank correlation coefficients (ρ) were calculated between the relative abundances of all bacterial genera and fungal genera. Only correlations with an absolute coefficient (|ρ|) ≥ 0.6 and a *p*-value < 0.05 after false discovery rate (FDR) correction using the Benjamini–Hochberg method were considered statistically significant and retained for network construction. This stringent threshold was chosen to focus on strong and robust inter-kingdom associations.

Network Visualization and Topological Analysis: The resulting correlation matrices were imported into Gephi (version 0.10) for visualization and topological analysis. The following conventions were applied: ①Nodes represent microbial genera; ②Node size is proportional to the mean relative abundance of the corresponding genus within the group; ③Node color and shape encode kingdom: bacterial genera are depicted as yellow circles, and fungal genera as green squares; ④Edges represent statistically significant correlations; ⑤Edge color indicates correlation direction: red edges denote positive correlations (ρ > 0), and blue edges denote negative correlations (ρ < 0).

Interpretation of Ecological States: The term “co-dysbiosis” is proposed to describe the network state observed in ARFC, characterized by a predominance of positive correlations and a loss of competitive (negative) interactions. This concept is used to hypothesize an ecological shift from a homeostatic, antagonism-rich network (HC) toward a simplified, cooperation-dominant network (ARFC), which may reflect reduced ecological resilience and facilitate disease-associated community consolidation.

## 3 Results

### 3.1 Altered gut fungal community structure without significant diversity changes in ARFC children

**Figure 1:**
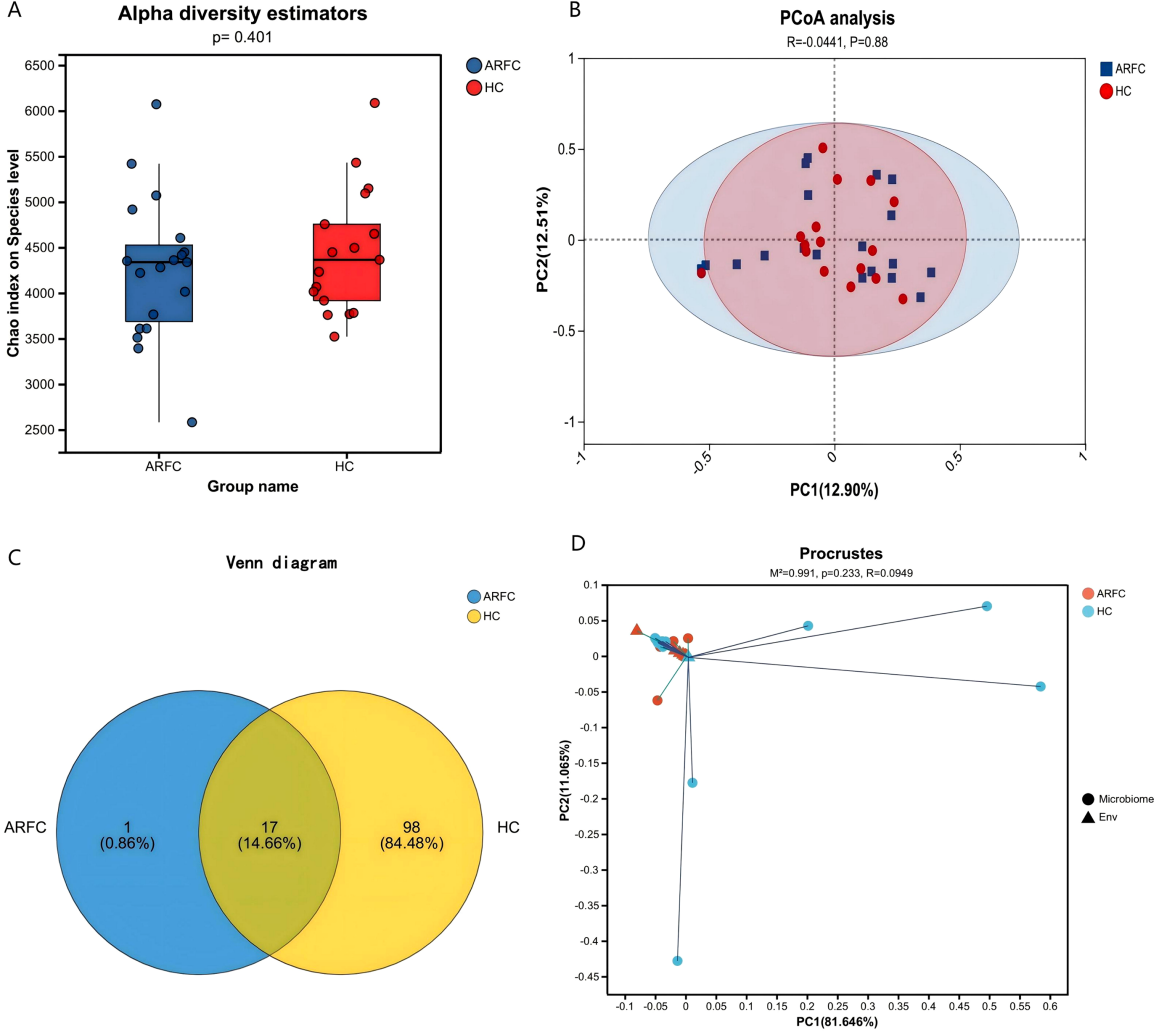
Gut fungal community diversity in children with allergic rhinitis and functional constipation (ARFC) and healthy controls (HC). **(A)** Chao1 index showing no significant difference in fungal richness between groups (Wilcoxon test, *p* = 0.401). **(B)** Principal coordinate analysis (PCoA) based on Bray–Curtis distances illustrating structural separation between ARFC and HC groups. **(C)** Venn diagram depicting the number of unique and shared fungal operational taxonomic units (OTUs) between groups. **(D)** Procrustes analysis indicating no significant overall configuration difference (M² = 0.991, *p* = 0.233). Sample sizes: n = 19 ARFC, n = 17 HC.

No significant alpha diversity differences were observed (Chao1, p = 0.401; **Figure 1A**). However, PCoA revealed partial structural separation (Bray–Curtis; **Figure 1B**). PERMANOVA was non-significant (R² = 0.0441, p = 0.88), likely due to inter-individual variation and limited sample size. HC contained 98 unique fungal OTUs versus one in ARFC (**Figure 1C**). Procrustes analysis showed no configuration difference (M² = 0.991, R = 0.0949, p = 0.233; **Figure 1D**).

### 3.2 Altered Fungal composition and multi-level biomarkers in ARFC

**Figure 2:**
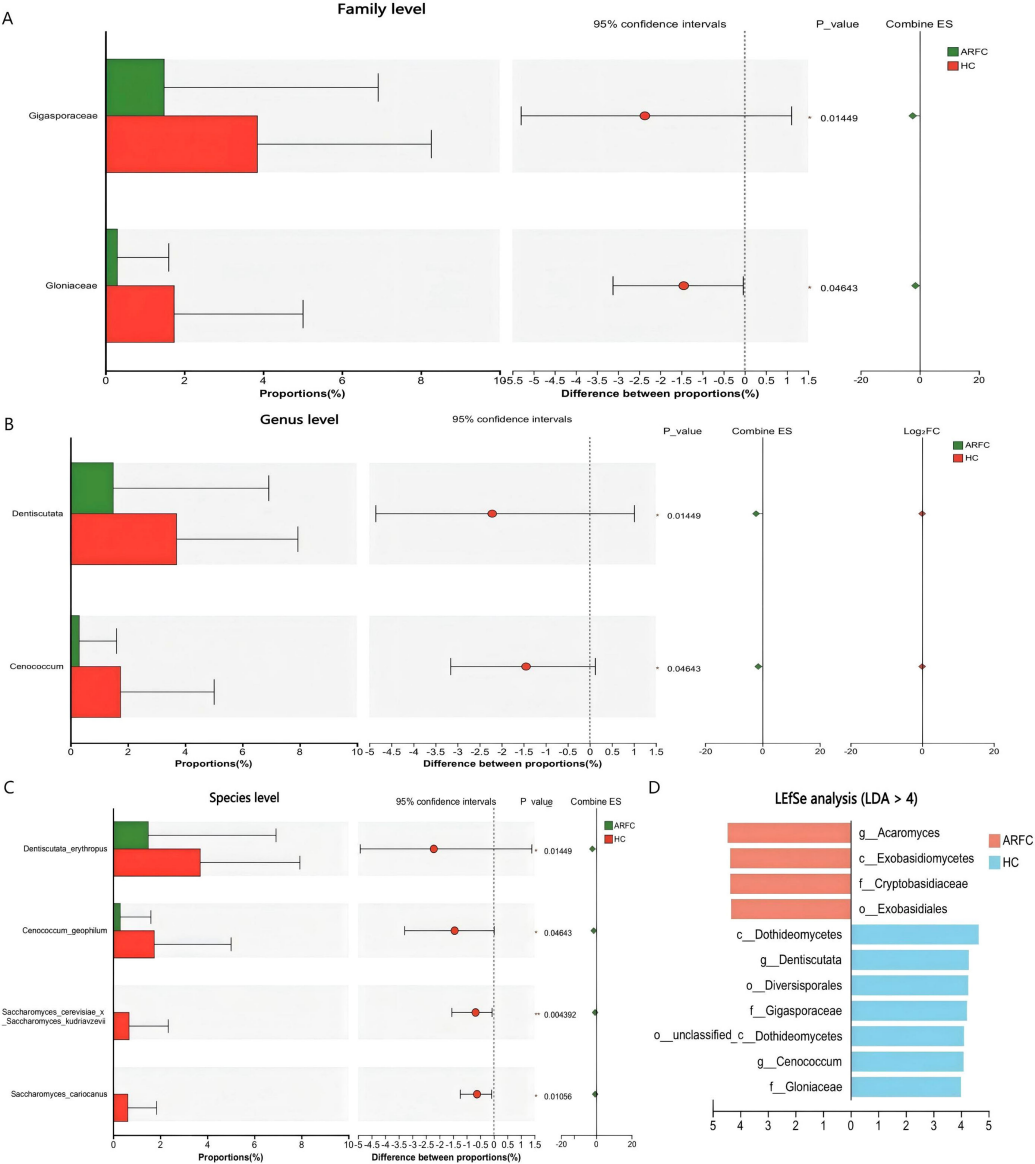
Differential abundance of fungal taxa at the family and genus levels between ARFC and HC groups. **(A)** Bar plot showing relative abundance of fungal families with significant differences (Wilcoxon test, *p* < 0.05). **(B)** Relative abundance of fungal genera significantly enriched in HC. **(C)** Box plots of selected fungal species exclusively detected in HC. **(D)** Linear discriminant analysis effect size (LEfSe) identifying fungal taxa enriched in HC (LDA score > 4). Data are presented as mean ± SD; *p-*values indicate statistical significance.

No phylum-level differences occurred (p > 0.05). At the family level, Gigasporaceae (HC: 3.85 ± 4.41% vs. ARFC: 1.49 ± 5.43%; p = 0.015) and Gloniaceae (HC: 1.74 ± 3.27% vs. ARFC: 0.30 ± 1.31%; p = 0.046) were enriched in HC (**Supplementary Table S2**; **Figure 2A**).

At the genus level, Dentiscutata (HC: 3.70 ± 4.24% vs. ARFC: 1.49 ± 5.43%; p = 0.015) and Cenococcum (HC: 1.74 ± 3.27% vs. ARFC: 0.30 ± 1.31%; p = 0.046) were depleted in ARFC (**Supplementary Table S3**; **Figure 2B**). Species-level depletion included Saccharomyces cerevisiae x S. kudriavzevii (HC: 0.67 ± 1.67% vs. ARFC: 0%; p = 0.004) and S. cariocanus(HC: 0.62 ± 1.22% vs. ARFC: 0%; p = 0.011) (**Supplementary Table S4**; **Figure 2C**).

LEfSe identified HC-enriched biomarkers (LDA >4): Glomeromycota taxa (e.g., Dentiscutata) and Ascomycota taxa (e.g., Cenococcum). ARFC showed enrichment in Basidiomycota classes (**Figure 2D**).

### 3.3 Key discriminators in random forest model

**Figure 3:**
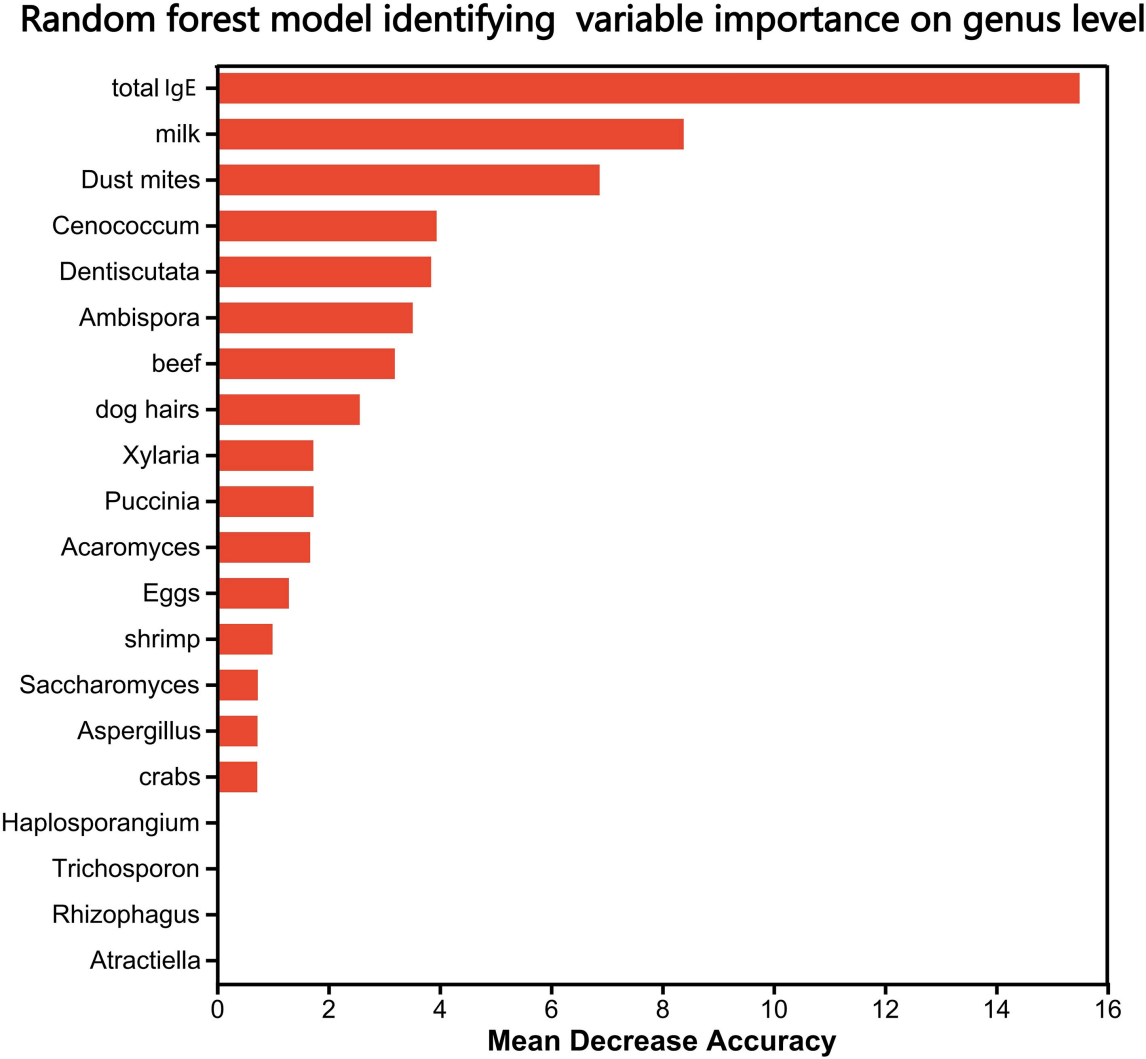
Random forest model identifying key fungal and clinical discriminators between ARFC and HC groups. Variable importance is ranked by MeanDecreaseGini. Total IgE was the most predictive clinical variable. Fungal genera including Cenococcum, Dentiscutata, and Ambispora exhibited high discriminatory power. Model performance was evaluated using 10-fold cross-validation (AUC and OOB error provided in methods).

The random forest model confirmed depleted genera (Cenococcum, Dentiscutata, Ambispora) as top discriminators (**Figure 3**). Total IgE was the most predictive clinical variable (MeanDecreaseGini = 15.5). Fungi outperformed some allergen-specific IgEs (e.g., *Cenococcum* importance = 3.95). Depleted genera (e.g., *Trichoderma*) exhibited negative importance scores.

### 3.4 Correlations between gut fungi and immunological parameters

**Figure 4:**
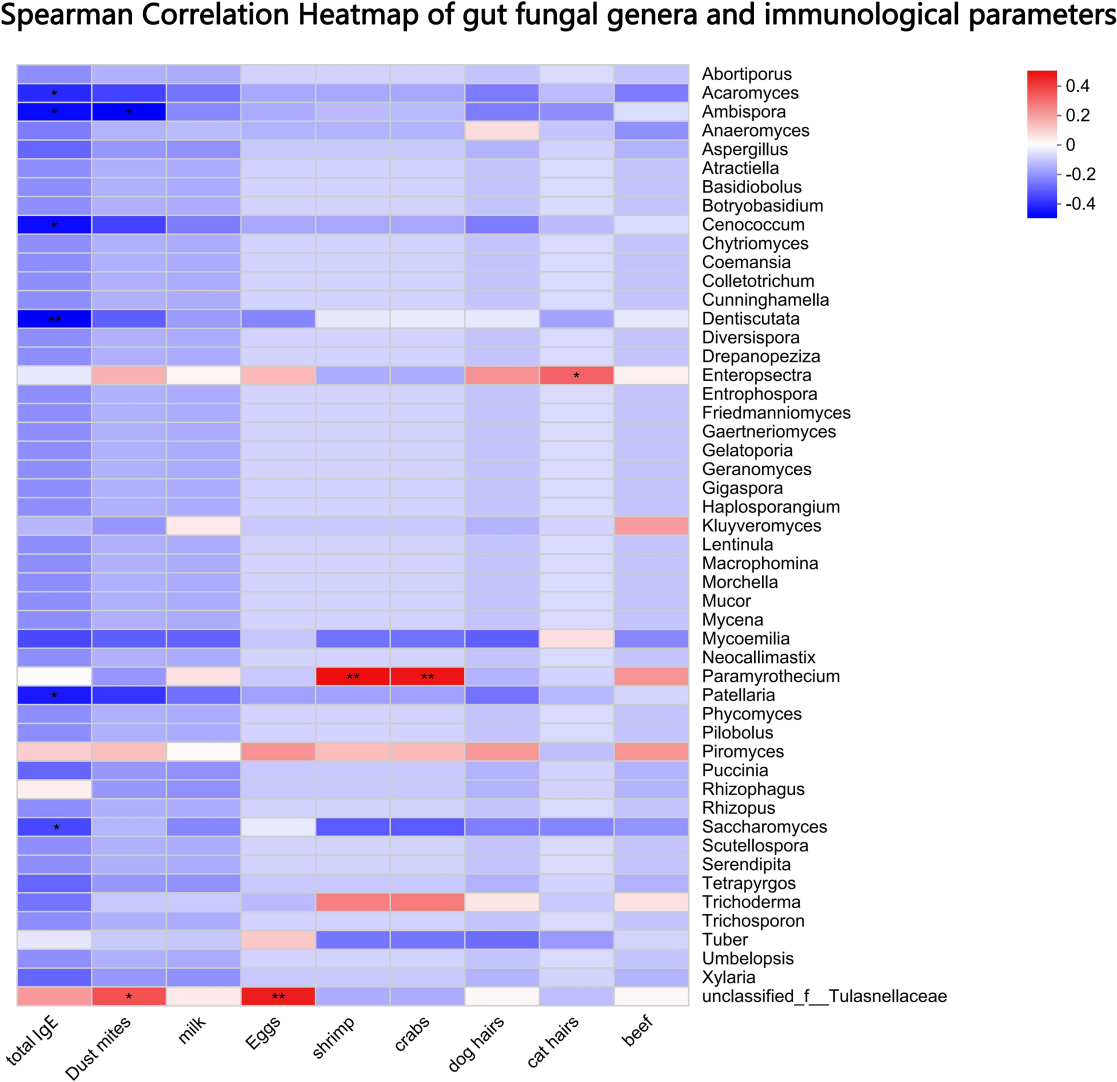
Spearman correlation analysis between gut fungal genera and immunological parameters. Heatmap displays correlation coefficients (ρ) between fungal abundances and total or allergen-specific IgE levels. Significant negative correlations were observed for Saccharomyces, Ambispora, Dentiscutata, and Cenococcum with total IgE and house dust mite-specific IgE. Only correlations with *p* < 0.05 are shown. * p < 0.05, ** p < 0.01.

Spearman correlation analysis revealed significant associations between gut fungal genera and immunological parameters (**Figure 4**): 1) Negative correlations: The abundances of *Saccharomyces* (ρ = -0.39), *Ambispora* (ρ = -0.40), *Dentiscutata* (ρ = -0.43), *Patellaria* (ρ = -0.37), *Acaromyces* (ρ = -0.34) and *Cenococcum* (ρ = -0.40) were significantly inversely correlated with total serum IgE, and specific IgE levels against house dust mites (*Ambispora* ρ = -0.42). 2) Positive correlation: The abundance of *Paramyrothecium* showed a positive correlation with specific IgE against shrimp (ρ = 0.50) and crab (ρ = 0.49); Tulasnellaceae with specific IgE against dust mites (ρ = 0.37) and eggs (ρ = 0.47). 3) No significant correlation: Most low-abundance fungal genera showed no significant correlation with the measured IgE levels.

### 3.5 No significant differences in predicted fungal functional profiles

**Figure 5:**
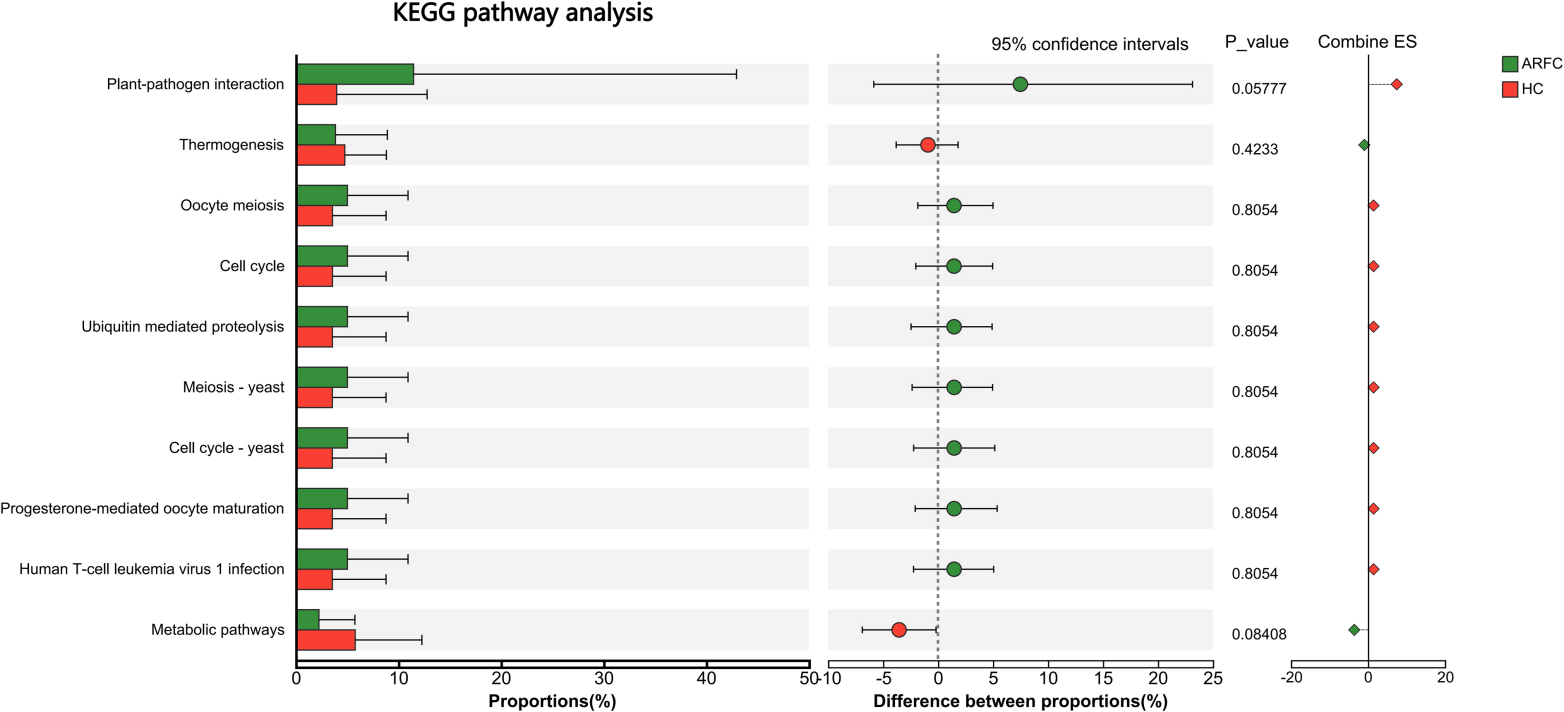
Predicted functional potential of the gut mycobiota based on KEGG pathway analysis. No significant differences were observed between ARFC and HC groups at any KEGG pathway level (Levels 1–3), as assessed by the ReporterScore algorithm (absolute score < |1.5|, not significant). Bar plots represent the relative abundance of KEGG pathways in each group.

Comparative analysis of the gut mycobiota’s functional potential via KEGG pathway enrichment showed no significant differences between the ARFC and HC groups at either Level 1, Level 2 and Level 3 categories (**Figure 5**), indicating that despite structural differences, the overall functional profile of the gut fungi remained relatively conserved in children with ARFC.

### 3.6 Comparative analysis of gut bacteriome-mycobiome interaction networks in ARFC and HC children

**Figure 6:**
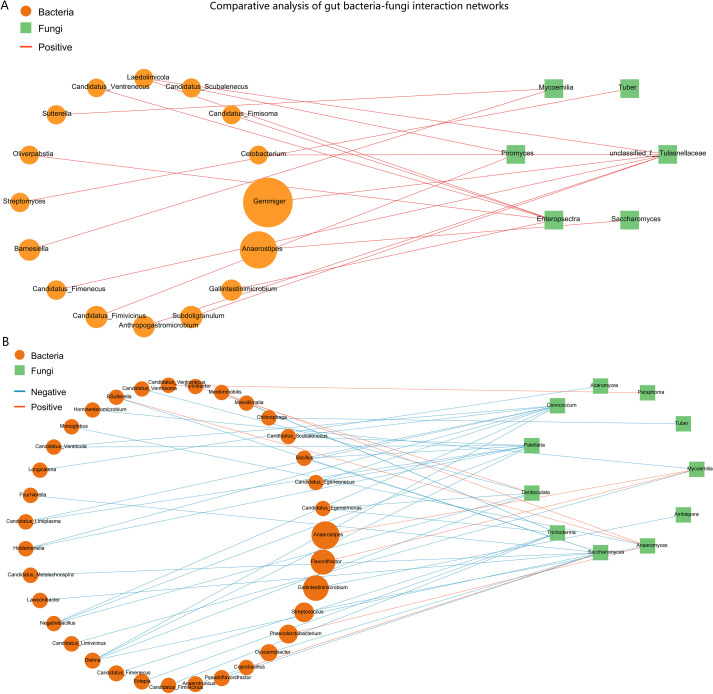
Comparative analysis of gut bacteria-fungi interaction networks in HC and ARFC children. Networks depict significant Spearman correlations (|r| ≥ 0.6, *p* < 0.05). Nodes: yellow circles (bacterial genera), green squares (fungal genera). Edges: red (positive correlations), blue (negative correlations). **(A)** HC network is characterized by numerous negative correlations, suggesting competitive interactions. **(B)** ARFC network is exclusively positive, indicating a shift toward cooperative interkingdom structure and potential “co-dysbiosis.”.

To elucidate the potential impact of the ARFC condition on the ecological relationships within the gut ecosystem, we constructed and compared bacterial–fungal correlation networks for the ARFC and HC groups. Bacterial abundance data were derived from the same metagenomic sequencing dataset used for mycobiota analysis, allowing integrated cross-kingdom network inference. This comparative analysis revealed profound differences in the structure and composition of the interkingdom networks between the two groups (**Figure 6**).

#### 3.6.1 Network in HC is characterized by prevalent negative interactions

The HC group network was complex and exhibited a high degree of negative correlations, which constituted the majority (44 out of 51 significant correlations, 86.3%) of all interactions. This suggests a network structure potentially dominated by competitive or antagonistic relationships under homeostasis. Notably, we screened the fungal contigs for putative antibiotic biosynthesis gene clusters (BGCs) using antiSMASH fungal mode (v7.0), which identified several biosynthetic pathways in fungal genera such as Cenococcum and Dentiscutata that are known to produce antifungal or antibacterial metabolites. These functional annotations lend ecological plausibility to the observed negative correlations, suggesting that fungal-derived antimicrobial activity may contribute to the competitive bacterial–fungal interactions observed in healthy controls. Key fungal genera, including *Saccharomyces, Patellaria, Cenococcum*, and *Dentiscutata*, acted as central hubs with multiple negative bacterial partners. For instance, the bacterium *Bacillus* was negatively correlated with *Saccharomyces* (r = -0.75, *p* = 0.0005), *Patellaria*, and *Cenococcum*. Similarly, the bacterium Dielma displayed significant negative correlations with four different fungal genera (*Saccharomyces, Dentiscutata, Patellaria, Cenococcum*). A limited number of positive correlations were observed in the HC network, such as between *Flavonifractor* and *Mycoemilia* (r = 0.74, *p* = 0.0006).

#### 3.6.2 Network in ARFC patients shifts towards an exclusively positive topology

In stark contrast, the interaction network in the ARFC group was exclusively composed of positive correlations (17 out of 17, 100%). This unanimous shift towards cooperative interactions indicates a fundamental restructuring of the gut ecosystem in ARFC. The ARFC network was less complex and centered around fewer fungal hubs. The unclassified Tulasnellaceae and the genus *Enteropsectra* emerged as the core nodes, forming positive correlations with seven and six bacterial partners, respectively. Notably, several bacterial genera involved in these cooperative clusters, such as *Anaerostipes* and *Subdoligranulum*, are known short-chain fatty acid (SCFA) producers. For example, *Anaerostipes* was positively correlated with *Saccharomyces* (r = 0.71, *p* = 0.0007) in ARFC, whereas it was linked to *Mycoemilia* (r = 0.66, *p* = 0.0039) in HC.

#### 3.6.3 Comparative summary

The direct comparison highlights a dramatic loss of negative interactions and a simplification of network architecture in ARFC patients. The HC network’s structure, rich in negative correlations, may reflect a robust, homeostatic ecosystem where microbial populations check and balance each other. Conversely, the ARFC network’s shift to a uniformly positive correlation profile suggests a state of “co-dysbiosis,” where formerly competitive relationships are replaced by cooperative ones, potentially facilitating the consolidation of a disease-associated microbial community and contributing to the pathophysiology of both allergic inflammation and gastrointestinal dysmotility.

## 4 Discussion

This study provides the first characterization of the gut mycobiota in ARFC children, revealing a significant depletion of specific immunomodulatory fungi—including *Cenococcum, Dentiscutata, Ambispora*, and *Saccharomyces*—in children with ARFC. These fungal taxa served as top discriminators in our machine learning model and exhibited significant inverse correlations with both total and allergen-specific IgE levels. Moreover, cross-kingdom network analysis uncovered a profound ecological restructuring in ARFC, characterized by a shift from a homeostatic, competition-rich network in healthy controls to an exclusively cooperative state. This unanimous shift towards positive correlations may reflect a loss of ecological resilience. Competitive interactions are fundamental for maintaining community stability and resisting perturbation (34, 35). In microbial ecology, such simplified, cooperation-dominant networks have been associated with ecosystem instability and reduced functional redundancy (36). We therefore propose the term “co-dysbiosis” to describe this altered state, which emphasizes the concurrent disruption of bacterial-fungal interkingdom interactions and the loss of competitive microbial relationships that typify a healthy gut ecosystem. We hypothesize that this simplified, cooperation-dominant network may represent a loss of ecological resilience, a concept that warrants validation in future, larger-scale ecological studies.

The depletion of these fungi likely contributes to ARFC pathogenesis via disrupted immune regulation. Fungi such as *Saccharomyces* and *Cenococcum* engage with host pattern-recognition receptors like Dectin-1, influencing the Th2/regulatory T cell (Treg) balance and IgE production (14, 19, 37). Their loss, combined with the observed “co-dysbiosis”, may impair the production of key immunomodulatory metabolites. For instance, our findings suggest that *Saccharomyces* species, which were depleted in ARFC, may contribute to intestinal barrier function and immune modulation, as previous studies have implicated this genus in metabolite production and Treg induction (14, 37). Similarly, the reduction in Glomeromycota fungi (e.g., *Dentiscutata, Ambispora*) could plausibly diminish the production of aryl hydrocarbon receptor (AhR) ligands, which are crucial for maintaining immune tolerance (13, 16). However, direct evidence for these specific mechanisms in our cohort requires further validation. Based on our correlations, we speculate that the depletion of these immunomodulatory fungi, coupled with the observed “co-dysbiosis”, may collectively disrupt systemic immune homeostasis, thereby potentially facilitating IgE sensitization in ARFC. Direct experimental evidence for this causal pathway is needed to confirm this model.

The significant inverse correlations between genera such as *Saccharomyces, Ambispora, Dentiscutata*, and *Cenococcum* with IgE levels support their immunoregulatory potential. For instance, Saccharomyces species are known to enhance short-chain fatty acid (SCFA) production, which promotes Treg differentiation and suppresses IgE responses (37, 38). Similarly, Glomeromycota-related taxa (e.g., *Dentiscutata, Ambispora*) may contribute to the production of aryl hydrocarbon receptor (AhR) ligands, which are crucial for maintaining immune tolerance and restraining Th2-driven IgE production (13, 16).

Beyond metabolite-mediated effects, fungi including *Cenococcum* can directly engage host pattern-recognition receptors such as Dectin-1. This interaction modulates the SYK–CARD9 signaling axis, which plays a fundamental role in shaping the Th2/Treg balance and controlling IgE production (28, 30). Although we did not directly measure SCFAs or AhR activity, the consistent depletion of these immunoregulatory fungi aligns with their established roles in systemic immune homeostasis, providing a mechanistic basis for the elevated IgE sensitization observed in ARFC children.

Although we found no significant differences in the predicted functional potential (KEGG pathways) of the gut mycobiota, this absence of metabolic signals should be interpreted cautiously. Functional redundancy within the fungal community or limitations in current fungal functional databases (12, 31, 39) may obscure genuine metabolic differences. Importantly, the structural and ecological alterations we observed could exert critical immunologic effects through non-metabolic mechanisms, particularly via direct fungal-host interactions through pattern-recognition receptors (28, 30). Fungal community structure, particularly the depletion of immunomodulatory taxa, can influence host immunity via pattern-recognition receptors (e.g., Dectin-1, TLRs) and downstream signaling cascades such as the SYK–CARD9 axis, which regulates inflammasome activation, Th2/Treg balance, and IgE production (19, 28, 30). This is supported by studies in other immune-mediated conditions where fungal dysbiosis alters mucosal immunity without concomitant shifts in metabolic pathways (40, 41). Thus, while functional redundancy or database limitations may obscure metabolic differences, the immunologic impact of structural dysbiosis remains evident.

Our comparative bacterial-fungal network analysis further underscores a fundamental ecological restructuring in ARFC. The healthy control network was characterized by numerous negative correlations, suggesting a stable, competitive ecosystem that may help maintain microbial diversity and prevent pathogen expansion (6, 34). In stark contrast, the ARFC network exhibited an exclusively positive correlation profile, indicating a collapse of these competitive restraints. This unanimous shift towards positive correlations may reflect a loss of ecological resilience, as competitive interactions are fundamental for maintaining community stability and resisting perturbation (35). In microbial ecology, such simplified, cooperation-dominant networks have been associated with ecosystem instability and reduced functional redundancy (36). This simplified, cooperative state, centered around new hub taxa like Tulasnellaceae, may facilitate the persistence of a disease-associated microbial community.

We propose the term “co-dysbiosis” to describe this altered state, which emphasizes the concurrent disruption of bacterial–fungal interkingdom interactions and the loss of competitive microbial relationships that typify a healthy gut ecosystem (34, 42). Importantly, while our bacterial data were derived from metagenomic reads, the consistent and dramatic shift from competitive to exclusively cooperative correlations suggests a fundamental ecological reorganization in ARFC. This simplified, cooperation-dominant network may reflect a loss of ecological resilience, a concept that warrants validation in future, larger-scale studies with matched multi-kingdom profiling (35, 36). Additionally, the identification of antibiotic biosynthesis gene clusters (BGCs) in fungal taxa such as Cenococcum and Dentiscutata—which were depleted in ARFC—suggests that these fungi may produce metabolites capable of modulating bacterial communities (43, 44). The loss of such fungi could therefore contribute to a “double dysbiosis”: not only a reduction in immunoregulatory fungi but also a disruption in fungal-mediated bacterial inhibition. This may further destabilize the gut ecosystem, shifting from a competitive, homeostatic network to a cooperative, dysbiotic state characterized by reduced microbial antagonism and potentially facilitating the emergence of disease-associated consortia (45, 46). This hypothesis aligns with the observed simplification of the ARFC network and underscores the interconnectedness of bacterial and fungal dysbiosis in ARFC pathogenesis.

In addition to immune dysregulation, the observed fungal dysbiosis and “co-dysbiosis” may also contribute to the gastrointestinal symptoms of ARFC, particularly constipation. Our network analysis revealed that in ARFC, several short-chain fatty acid (SCFA)-producing bacterial genera (e.g., *Anaerostipes, Subdoligranulum*) formed cooperative clusters with fungi such as Tulasnellaceae and Enteropsectra. While SCFAs like butyrate are generally promotive of gut motility, their net effect depends on the broader ecological context and interacting microbial consortia (47). The shift from a competitive to an exclusively cooperative network (“co-dysbiosis”) may alter the overall metabolic output of the gut ecosystem. This could facilitate the overgrowth or altered activity of bacterial taxa previously linked to constipation, such as specific Clostridium clusters involved in bile acid transformation (48) or mucin-degrading bacteria that affect mucosal turnover and stool consistency (49). For instance, reductions in *Bacteroides* and increases in certain *Firmicutes* have been associated with slowed colonic transit (49). The loss of fungal-derived inhibitory metabolites (e.g., from depleted *Cenococcum*) might remove a natural check on such bacteria, thereby contributing to dysmotility. Thus, “co-dysbiosis” may represent an ecological driver linking mycobiota alterations to both immune (IgE sensitization) and motor (constipation) components of ARFC. Furthermore, diet—a key modulator of gut microbiota—was not systematically assessed in this pilot study, but future investigations should evaluate dietary patterns (e.g., fiber intake, fermentable carbohydrates) as potential confounders or effect modifiers linking mycobiota dysbiosis, bacterial community structure, and stool characteristics in ARFC (50, 51).

In summary, this study provides the first multi-faceted characterization of gut mycobiota dysbiosis in children with ARFC, encompassing the depletion of specific immunomodulatory fungi and a profound ecological restructuring of bacterial-fungal networks from a competitively dominant state to an exclusively cooperative one, termed “co-dysbiosis.” These alterations are significantly correlated with elevated serum IgE levels. Based on these interconnected findings, we propose an integrative mechanistic hypothesis (as summarized in the Graphical Abstract): In the healthy state, a diverse mycobiota, including genera such as *Cenococcum* and *Dentiscutata*, contributes to immune homeostasis by engaging host pattern-recognition receptors (e.g., Dectin-1) and facilitating the production of immunomodulatory metabolites (e.g., SCFAs), thereby suppressing IgE responses. In ARFC, the depletion of these protective fungi, coupled with the simplified, cooperation-dominant network architecture of “co-dysbiosis,” collectively impairs intestinal immunoregulation. This disruption may facilitate a Th2-skewed immune response and IgE sensitization via the putative “gut–fungi–nose axis,” ultimately contributing to the comorbid clinical manifestations of allergic rhinitis and constipation. While this hypothesis requires validation in future studies, it offers a novel, mycobiota-centric framework for understanding ARFC pathogenesis.

Several limitations should be acknowledged. First, the cross-sectional design precludes causal inference about the relationship between mycobiota alterations and disease development. Second, the sample size, appropriate for a pilot investigation, inevitably limits the statistical power for detecting subtle associations. However, this design was instrumental in generating the first high-dimensional map of the mycobiota in ARFC and identifying robust, large-effect-size signals (e.g., the depletion of key genera and the striking network shift) that provide a clear rationale and specific hypotheses for future, larger-scale studies. Third, our metagenomic approach for mycobiota characterization employed VirSorter2 and DeepVirFinder to maximize sensitivity for detecting diverse fungal sequences. While we applied stringent *post-hoc* filters (e.g., CheckV, alignment against host and bacterial genomes), this method may carry a risk of misclassifying non-fungal elements. Future studies should validate these findings using complementary approaches such as ITS amplicon sequencing or fungal-specific marker gene analysis. Fourth, while bacterial abundance data were extracted from the same metagenomic libraries for network construction—ensuring sample-level integration—the sequencing depth and analytical pipeline were primarily optimized for fungal sequence recovery. Consequently, metagenomic-based bacterial profiling, while providing a direct cross-kingdom dataset, may offer lower taxonomic resolution for rare or low-abundance bacterial taxa compared to targeted 16S rRNA gene sequencing. This could potentially affect the detection of subtle interkingdom associations involving these minority bacterial populations. Future studies employing matched, deep shotgun sequencing optimized for both kingdoms, or complementary 16S rRNA sequencing, would help validate the observed network topology and better resolve the specific bacterial partners within the “co-dysbiosis” state. Fifth, we did not quantify fungal biomass using methods such as qPCR targeting fungal rRNA genes, which would have helped confirm that the lack of alpha diversity differences reflects biological reality rather than technical variation in sequencing depth. Finally, taxonomic annotation remains constrained by the underrepresentation of fungal genomes in public databases, particularly for environmental and plant-associated fungi that may transiently inhabit the human gut.

Future studies should employ larger, longitudinal cohorts to validate these findings and explore causality. Integrated multi-omics approaches—combining metagenomics, metatranscriptomics, and metabolomics—are needed to elucidate the functional mechanisms of specific fungi and their interactions with bacteria. Furthermore, *in vivo* and *in vitro* models are essential to confirm the immunomodulatory roles of the depleted fungi and their potential as therapeutic targets (8, 19).

Our pilot study unveils a multi-faceted dysbiosis of the gut mycobiota in ARFC, encompassing taxonomic depletion, profound ecological restructuring, and significant immunological correlations. To synthesize these interconnected findings and their broader implications, we present a conceptual summary in **Table 2**.

**Table 2:** Conceptual summary of gut mycobiota alterations, ecological restructuring, and immunological correlates in children with comorbid allergic rhinitis and functional constipation (ARFC).

Aspect	Key observations in ARFC vs. HC	Proposed immunological/ecological mechanisms	Novel concepts/hypotheses generated	Clinical & research implications
Taxonomic Dysbiosis	Depletion of immunomodulatory fungi (e.g., *Cenococcum, Dentiscutata, Ambispora, Saccharomyces*).	1. Loss of immunoregulation: Reduced engagement of PRRs (e.g., Dectin-1/SYK-CARD9), potentially skewing Th2/Treg balance. 2. Diminished metabolite production: Possible reduction in SCFAs and AhR ligands, crucial for immune tolerance and barrier integrity.	“Protective Mycobiota Cluster” Hypothesis: A consortium of specific fungi acts synergistically to maintain gut immune homeostasis; their collective loss disinhibits IgE sensitization.	Therapeutic targets: These fungi or their metabolites are candidates for probiotics/postbiotics aimed at restoring immune balance in ARFC.
Ecological Restructuring	Shift from a complex, competition-rich bacterial-fungal network (HC) to a simplified, exclusively cooperative network (ARFC).	1. Loss of ecological resilience: Collapse of competitive interactions reduces community stability and functional redundancy. 2.Fungal-mediated inhibition loss: Depleted fungi (e.g., *Cenococcum*) may have produced antimicrobial metabolites (BGCs), whose absence reduces checks on bacterial partners.	“Co-dysbiosis”: A disease state characterized by pervasive positive cross-kingdom correlations, indicating a breakdown of homeostatic microbial antagonism and potential consolidation of a pro-inflammatory consortium.	Diagnostic biomarker: Network topology (competition vs. cooperation ratio) could be a novel ecological indicator of ARFC.
Functional & Clinical Correlates	1. Strong inverse correlations between depleted fungi and serum IgE levels. 2.No significant difference in predicted KEGG metabolic pathways.	Immune dysregulation may be driven more by structural recognition of fungi (via PRRs) than by bulk metabolic output, highlighting non-metabolic immunomodulation.	“Structure Over Function” Paradigm: In the gut mycobiota, the presence/absence and interaction patterns of key taxa may be more immunologically critical than aggregate metabolic potential.	Future studies should focus on host-fungal interactomics and trans-kingdom signaling, not just cataloging metabolic genes.
Limitations & Future Research	Pilot study with cross-sectional design, limited sample size, and methodological focus on fungal DNA detection.	Current findings establish correlation; causality and precise mechanisms require further validation.	integrated “Gut-Fungi-Nose Axis”: Provides a framework to investigate how intestinal fungal dysbiosis influences remote allergic inflammation in the upper airways.	Future directions: 1) Longitudinal cohorts; 2) Multi-omics (meta-transcriptomics, metabolomics); 3) Gnotobiotic models to test causality of depleted fungi; 4) Assessment of dietary confounders.

## 5 Conclusion

This pilot study demonstrates gut mycobiota dysbiosis in ARFC children, marked by depletion of beneficial fungi and a shift to “co-dysbiosis.” These alterations correlate with IgE sensitization and may underlie gut–nose axis dysregulation. Despite structural changes, functional metabolic potential was conserved. The mycobiota emerges as a critical element in ARFC pathogenesis, warranting larger-scale validation.

## Data Availability

The raw metagenomic sequencing data generated in this study have been deposited in the NCBI Sequence Read Archive (SRA) under accession number PRJNA1279672. These data will be made publicly accessible upon publication via the following link: https://www.ncbi.nlm.nih.gov/sra/PRJNA1279672.

## References

[1] Yamaguchi T Nomura A Matsubara A Hisada T Tamada Y Mikami T . Effect of gut microbial composition and diversity on major inhaled allergen sensitization and onset of allergic rhinitis. Allergol Int. (2023) 72:135–42. doi: 10.1016/j.alit.2022.06.005, PMID: 35850746

[2] Kallio S Jian C Korpela K Kukkonen AK Salonen A Savilahti E . Early-life gut microbiota associates with allergic rhinitis during 13-year follow-up in a Finnish probiotic intervention cohort. Microbiol Spectr. (2024) 12:e0413523. doi: 10.1128/spectrum.04135-23, PMID: 38687061 PMC11324021

[3] Wu MC Jan MS Chiou JY Wang YH Wei JC . Constipation might be associated with risk of allergic rhinitis: A nationwide population-based cohort study. PloS One. (2020) 15:e0239723. doi: 10.1371/journal.pone.0239723, PMID: 33006996 PMC7531808

[4] Lee MH Wu MC Wang YH Wei JC . Maternal constipation is associated with allergic rhinitis in the offspring: A nationwide retrospective cohort study. PloS One. (2023) 18:e0292594. doi: 10.1371/journal.pone.0292594, PMID: 37797074 PMC10553815

[5] Ho SW Lin CP Ku MS . The impact of allergic rhinitis on gastrointestinal disorders among young adults. J Eval Clin Pract. (2020) 26:242–7. doi: 10.1111/jep.13108, PMID: 30773746

[6] Zhou MS Zhang B Gao ZL Zheng RP . Altered diversity and composition of gut microbiota in patients with allergic rhinitis. Microb Pathog. (2021) 161:105272. doi: 10.1016/j.micpath.2021.105272, PMID: 34740809

[7] Zhang S Wang R Li D Zhao L Zhu L . Role of gut microbiota in functional constipation. Gastroenterol Rep (Oxf). (2021) 9:392–401. doi: 10.1093/gastro/goab035, PMID: 34733524 PMC8560038

[8] Wang C Liu H Li X Kong W Wu H Huang C . Multiomics technology reveals the changes in gut microbiota to stimulate aromatic amino acid metabolism in children with allergic rhinitis and constipation. Front Allergy. (2025) 6:1562832. doi: 10.3389/falgy.2025.1562832, PMID: 40416823 PMC12098344

[9] Salgaço MK Perina NP Tomé TM Mosquera EMB Lazarini T Sartoratto A . Probiotic infant cereal improves children’s gut microbiota: Insights using the Simulator of Human Intestinal Microbial Ecosystem (SHIME^®^). Food Res Int. (2021) 143:110292. doi: 10.1016/j.foodres.2021.110292, PMID: 33992391

[10] Chiu CY Cheng ML Chiang MH Kuo YL Tsai MH Chiu CC . Gut microbial-derived butyrate is inversely associated with IgE responses to allergens in childhood asthma. Pediatr Allergy Immunol. (2019) 30:689–97. doi: 10.1111/pai.13096, PMID: 31206804

[11] Botschuijver S Roeselers G Levin E Jonkers DM Welting O Heinsbroek SEM . Intestinal fungal dysbiosis is associated with visceral hypersensitivity in patients with irritabl e bowel syndrome and rats. Gastroenterology. (2017) 153:1026–39. doi: 10.1053/j.gastro.2017.06.004, PMID: 28624575

[12] Iliev ID Cadwell K . Effects of intestinal fungi and viruses on immune responses and inflammatory bowel diseases. Gastroenterology. (2021) 160:1050–66. doi: 10.1053/j.gastro.2020.06.100, PMID: 33347881 PMC7956156

[13] Huang H Wang Q Yang Y Zhong W He F Li J . The mycobiome as integral part of the gut microbiome: crucial role of symbiotic fungi in health and disease. Gut Microbes. (2024) 16:2440111. doi: 10.1080/19490976.2024.2440111, PMID: 39676474 PMC11651280

[14] Wu X Xia Y He F Zhu C Ren W . Intestinal mycobiota in health and diseases: from a disrupted equilibrium to clinical opportunities. Microbiome. (2021) 9:60. doi: 10.1186/s40168-021-01024-x, PMID: 33715629 PMC7958491

[15] Li XV Leonardi I Iliev ID . Gut mycobiota in immunity and inflammatory disease. Immunity. (2019) 50:1365–79. doi: 10.1016/j.immuni.2019.05.023, PMID: 31216461 PMC6585451

[16] Zhang F Aschenbrenner D Yoo JY Zuo T . The gut mycobiome in health, disease, and clinical applications in association with the gut bacterial microbiome assembly. Lancet Microbe. (2022) 3:e969–83. doi: 10.1016/s2666-5247(22)00203-8, PMID: 36182668

[17] Leonardi I Li X Semon A Li D Doron I Putzel G . CX3CR1(+) mononuclear phagocytes control immunity to intestinal fungi. Science. (2018) 359:232–6. doi: 10.1126/science.aao1503, PMID: 29326275 PMC5805464

[18] Yan Q Li S Yan Q Huo X Wang C Wang X . A genomic compendium of cultivated human gut fungi characterizes the gut mycobiome and its relevance to common diseases. Cell. (2024) 187:2969–2989.e2924. doi: 10.1016/j.cell.2024.04.043, PMID: 38776919

[19] Glatthardt T van Tilburg Bernardes E Arrieta MC . The mycobiome in atopic diseases: Inducers and triggers. J Allergy Clin Immunol. (2023) 152:1368–75. doi: 10.1016/j.jaci.2023.10.006, PMID: 37865199

[20] Li X Leonardi I Semon A Doron I Gao IH Putzel GG . Response to fungal dysbiosis by gut-resident CX3CR1(+) mononuclear phagocytes aggravates allergic airway disease. Cell Host Microbe. (2018) 24:847–856.e844. doi: 10.1016/j.chom.2018.11.003, PMID: 30503509 PMC6292739

[21] Wang Y He F Liu B Wu X Han Z Wang X . Interaction between intestinal mycobiota and microbiota shapes lung inflammation. Imeta. (2024) 3:e241. doi: 10.1002/imt2.241, PMID: 39429884 PMC11487552

[22] Barcik W Boutin RCT Sokolowska M Finlay BB . The role of lung and gut microbiota in the pathology of asthma. Immunity. (2020) 52:241–55. doi: 10.1016/j.immuni.2020.01.007, PMID: 32075727 PMC7128389

[23] Skalski JH Limon JJ Sharma P Gargus MD Nguyen C Tang J . Expansion of commensal fungus Wallemia mellicola in the gastrointestinal mycobiota enhances the severity of allergic airway disease in mice. PloS Pathog. (2018) 14:e1007260. doi: 10.1371/journal.ppat.1007260, PMID: 30235351 PMC6147580

[24] Shibuya A Shibuya K . Exploring the gut fungi-lung allergy axis. Cell Host Microbe. (2018) 24:755–7. doi: 10.1016/j.chom.2018.11.012, PMID: 30543774

[25] Zhu L Wu Y Lin C Tang L Yu B Wan W . Dynamic microbial shifts and signatures of long-term remission in allergic rhinitis after an herbal formula treatment. Front Immunol. (2021) 12:774966. doi: 10.3389/fimmu.2021.774966, PMID: 34745150 PMC8569905

[26] Zhang P Zhou X Tan H Jian F Jing Z Wu H . Microbial signature of intestine in children with allergic rhinitis. Front Microbiol. (2023) 14:1208816. doi: 10.3389/fmicb.2023.1208816, PMID: 37560527 PMC10408450

[27] Hu Y Zhang R Li J Wang H Wang M Ren Q . Association between gut and nasal microbiota and allergic rhinitis: A systematic review. J Asthma Allergy. (2024) 17:633–51. doi: 10.2147/jaa.S472632, PMID: 39006241 PMC11246088

[28] Kanj AN Kottom TJ Schaefbauer KJ Choudhury M Limper AH Skalski JH . Dysbiosis of the intestinal fungal microbiota increases lung resident group 2 innate lymphoid cells and is associated with enhanced asthma severity in mice and humans. Respir Res. (2023) 24:144. doi: 10.1186/s12931-023-02422-5, PMID: 37259076 PMC10230676

[29] Zheng F Yang Y Lu G Tan JS Mageswary U Zhan Y . Metabolomics insights into gut microbiota and functional constipation. Metabolites. (2025) 15:269. doi: 10.3390/metabo15040269, PMID: 40278398 PMC12029362

[30] Malik A Sharma D Malireddi RKS Guy CS Chang TC Olsen SR . SYK-CARD9 signaling axis promotes gut fungi-mediated inflammasome activation to restrict colitis and colon cancer. Immunity. (2018) 49:515–530.e515. doi: 10.1016/j.immuni.2018.08.024, PMID: 30231985 PMC6541497

[31] Li J Chen D Yu B He J Zheng P Mao X . Fungi in gastrointestinal tracts of human and mice: from community to functions. Microb Ecol. (2018) 75:821–9. doi: 10.1007/s00248-017-1105-9, PMID: 29110065

[32] Hyams JS Di Lorenzo C Saps M Shulman RJ Staiano A van Tilburg M . Functional disorders: children and adolescents. Gastroenterology. (2016) S0016-5085(16)00181–5. doi: 10.1053/j.gastro.2016.02.015, PMID: 27144632

[33] Cheng L . Revising allergic rhinitis guidelines to standardize clinical diagnosis and treatment. Zhonghua Er Bi Yan Hou Tou Jing Wai Ke Za Zhi. (2022) 57:413–7. doi: 10.3760/cma.j.cn115330-20220325-00134, PMID: 35527431

[34] Maas E Penders J Venema K . Fungal-bacterial interactions in the human gut of healthy individuals. J Fungi (Basel). (2023) 9:139. doi: 10.3390/jof9020139, PMID: 36836254 PMC9965947

[35] Coyte KZ Schluter J Foster KR . The ecology of the microbiome: Networks, competition, and stability. Science. (2015) 350:663–6. doi: 10.1126/science.aad2602, PMID: 26542567

[36] Faust K Raes J . Microbial interactions: from networks to models. Nat Rev Microbiol. (2012) 10:538–50. doi: 10.1038/nrmicro2832, PMID: 22796884

[37] Wang H Wu H Li KD Wang YY Huang RG Du YJ . Intestinal fungi and systemic autoimmune diseases. Autoimmun Rev. (2023) 22:103234. doi: 10.1016/j.autrev.2022.103234, PMID: 36423833

[38] Zhang H Wei Y Jia H Chen D Tang X Wang J . Immune activation of characteristic gut mycobiota Kazachstania pintolopesii on IL-23/IL-17R signaling in ankylosing spondylitis. Front Cell Infect Microbiol. (2022) 12:1035366. doi: 10.3389/fcimb.2022.1035366, PMID: 36605130 PMC9808786

[39] Narunsky-Haziza L Sepich-Poore GD Livyatan I Asraf O Martino C Nejman D . Pancancer analyses reveal cancer-type-specific fungal ecologies and bacteriome interactions. Cell. (2022) 185:3789–3806.e17. doi: 10.1016/j.cell.2022.09.005, PMID: 36179670 PMC9567272

[40] Carlson SL Mathew L Savage M Kok K Lindsay JO Munro CA . Mucosal immunity to gut fungi in health and inflammatory bowel disease. J Fungi (Basel). (2023) 9:1105. doi: 10.3390/jof9111105, PMID: 37998910 PMC10672531

[41] Kiran NS Chatterjee A Yashaswini C Deshmukh R Alsaidan OA Bhattacharya S . The gastrointestinal mycobiome in inflammation and cancer: unraveling fungal dysbiosis, pathogenesis, and therapeutic potential. Med Oncol. (2025) 42:195. doi: 10.1007/s12032-025-02761-x, PMID: 40323477

[42] Sam QH Chang MW Chai LY . The fungal mycobiome and its interaction with gut bacteria in the host. Int J Mol Sci. (2017) 18:330. doi: 10.3390/ijms18020330, PMID: 28165395 PMC5343866

[43] Baral B Akhgari A Metsä-Ketelä M . Activation of microbial secondary metabolic pathways: Avenues and challenges. Synth Syst Biotechnol. (2018) 3:163–78. doi: 10.1016/j.synbio.2018.09.001, PMID: 30345402 PMC6190515

[44] Nenciarini S Cavalieri D . Immunomodulatory potential of fungal extracellular vesicles: insights for therapeutic applications. Biomolecules. (2023) 13:1487. doi: 10.3390/biom13101487, PMID: 37892168 PMC10605264

[45] Tipton L Müller CL Kurtz ZD Huang L Kleerup E Morris A . Fungi stabilize connectivity in the lung and skin microbial ecosystems. Microbiome. (2018) 6:12. doi: 10.1186/s40168-017-0393-0, PMID: 29335027 PMC5769346

[46] van Tilburg Bernardes E Pettersen VK Gutierrez MW Laforest-Lapointe I Jendzjowsky NG Cavin JB . Intestinal fungi are causally implicated in microbiome assembly and immune development in mice. Nat Commun. (2020) 11:2577. doi: 10.1038/s41467-020-16431-1, PMID: 32444671 PMC7244730

[47] Saito Y Sato T Nomoto K Tsuji H . Identification of phenol- and p-cresol-producing intestinal bacteria by using media supplemented with tyrosine and its metabolites. FEMS Microbiol Ecol. (2018) 94:fiy125. doi: 10.1093/femsec/fiy125, PMID: 29982420 PMC6424909

[48] Zhao L Yang W Chen Y Huang F Lu L Lin C . A Clostridia-rich microbiota enhances bile acid excretion in diarrhea-predominant irrita ble bowel syndrome. J Clin Invest. (2020) 130:438–50. doi: 10.1172/JCI130976, PMID: 31815740 PMC6934182

[49] Roager HM Hansen LBS Bahl MI Frandsen HL Carvalho V Gøbel RJ . Colonic transit time is related to bacterial metabolism and mucosal turnover in the gut. Nat Microbiol. (2016) 1:16093. doi: 10.1038/nmicrobiol.2016.93, PMID: 27562254

[50] Sonnenburg ED Smits SA Tikhonov M Higginbottom SK Wingreen NS Sonnenburg JL . Diet-induced extinctions in the gut microbiota compound over generations. Nature. (2016) 529:212–5. doi: 10.1038/nature16504, PMID: 26762459 PMC4850918

[51] Asnicar F Berry SE Valdes AM Nguyen LH Piccinno G Drew DA . Microbiome connections with host metabolism and habitual diet from 1,098 deeply phenotyped individuals. Nat Med. (2021) 27:321–32. doi: 10.1038/s41591-020-01183-8, PMID: 33432175 PMC8353542

