# Supplementary material for: Gut mycobiota dysbiosis and an emergent state of “co-dysbiosis” are associated with IgE sensitization in children with comorbid allergic rhinitis and constipation
Source: Front Immunol. 2026 Jan 23;16:1745580. doi: 10.3389/fimmu.2025.1745580 (PMC12876214; doi:10.3389/fimmu.2025.1745580)
Supplement: Supplementary file 1 [file Table1.docx]

|  | rawdata | | | | | | cleandata | | | | | | splicing result | | | | | |
| --- | --- | --- | --- | --- | --- | --- | --- | --- | --- | --- | --- | --- | --- | --- | --- | --- | --- | --- |
| Sample | ReadsNum | BasesNum | Average Length | Raw Q20(%) | Raw Q30(%) | Raw GC content(%) | ReadsNum | BasesNum | Average Length | Clean Q20(%) | Clean Q30(%) | Clean GC content(%) | Contigs | Contigs bases(bp) | N50 (bp) | N90 (bp) | Max(bp) | Min (bp) |
| ARFC1 | 67759040 | 10163856000 | 150 | 0.9934 | 0.9786 | 0.4784 | 67349406 | 10062952656 | 149 | 0.995 | 0.9816 | 0.4769 | 95039 | 175208823 | 3528 | 646 | 336738 | 500 |
| ARFC10 | 89761878 | 13464281700 | 150 | 0.9935 | 0.9788 | 0.4628 | 89202478 | 13366054712 | 149 | 0.9949 | 0.9815 | 0.4615 | 97447 | 220938715 | 5870 | 723 | 547799 | 500 |
| ARFC11 | 87563024 | 13134453600 | 150 | 0.9929 | 0.9774 | 0.4906 | 86888990 | 12957376742 | 149 | 0.9949 | 0.9813 | 0.4886 | 122454 | 256940922 | 5547 | 689 | 377527 | 500 |
| ARFC12 | 84542952 | 12681442800 | 150 | 0.9934 | 0.9785 | 0.4863 | 84020042 | 12555982715 | 149 | 0.995 | 0.9815 | 0.4848 | 73094 | 159361990 | 4806 | 727 | 263435 | 500 |
| ARFC13 | 72496610 | 10874491500 | 150 | 0.9932 | 0.9775 | 0.4891 | 72079110 | 10782988034 | 149 | 0.9946 | 0.9802 | 0.4878 | 97908 | 225617979 | 5534 | 743 | 394014 | 500 |
| ARFC14 | 92961832 | 13944274800 | 150 | 0.9932 | 0.9781 | 0.4832 | 92296292 | 13787258087 | 149 | 0.995 | 0.9816 | 0.4813 | 154190 | 294188769 | 3882 | 670 | 611043 | 500 |
| ARFC15 | 94823654 | 14223548100 | 150 | 0.9916 | 0.975 | 0.4259 | 93131232 | 13903809187 | 149 | 0.9949 | 0.9816 | 0.4197 | 82994 | 170416974 | 4586 | 688 | 339697 | 500 |
| ARFC16 | 85465890 | 12905349390 | 151 | 0.9917 | 0.9632 | 0.4495 | 84789558 | 12782289118 | 150 | 0.9942 | 0.9678 | 0.4483 | 52838 | 130602846 | 11184 | 726 | 405832 | 500 |
| ARFC17 | 84227890 | 12718411390 | 151 | 0.9906 | 0.9595 | 0.4673 | 83438838 | 12578038325 | 150 | 0.9936 | 0.9652 | 0.466 | 111915 | 222399121 | 4310 | 681 | 508365 | 500 |
| ARFC18 | 84037698 | 12689692398 | 151 | 0.9911 | 0.961 | 0.4587 | 83337892 | 12555162558 | 150 | 0.9938 | 0.9661 | 0.4573 | 108425 | 236094106 | 5171 | 711 | 368967 | 500 |
| ARFC19 | 80894892 | 12215128692 | 151 | 0.9918 | 0.9628 | 0.4478 | 80303258 | 12108278201 | 150 | 0.9941 | 0.9671 | 0.4468 | 66106 | 176986551 | 7518 | 830 | 402303 | 500 |
| ARFC2 | 93799598 | 14069939700 | 150 | 0.9932 | 0.9778 | 0.4646 | 92951468 | 13905585600 | 149 | 0.9949 | 0.9813 | 0.4621 | 94673 | 208289746 | 7746 | 682 | 457415 | 500 |
| ARFC3 | 79129658 | 11869448700 | 150 | 0.9919 | 0.9756 | 0.4178 | 77731900 | 11615766844 | 149 | 0.995 | 0.9818 | 0.4114 | 27370 | 60843595 | 5386 | 713 | 492650 | 500 |
| ARFC4 | 103309138 | 15496370700 | 150 | 0.9933 | 0.9785 | 0.4742 | 102513794 | 15330337526 | 149 | 0.995 | 0.9818 | 0.4723 | 194280 | 344479600 | 2962 | 650 | 675698 | 500 |
| ARFC5 | 89172784 | 13375917600 | 150 | 0.9933 | 0.9781 | 0.4662 | 88515306 | 13239365238 | 149 | 0.9949 | 0.9813 | 0.4642 | 76790 | 206007605 | 9373 | 793 | 575159 | 500 |
| ARFC6 | 82801394 | 12420209100 | 150 | 0.993 | 0.9769 | 0.4775 | 82241562 | 12299134278 | 149 | 0.9946 | 0.98 | 0.4756 | 84095 | 159338752 | 4375 | 648 | 556955 | 500 |
| ARFC7 | 97476844 | 14621526600 | 150 | 0.9931 | 0.9781 | 0.4545 | 96649694 | 14439747522 | 149 | 0.995 | 0.9819 | 0.4522 | 82489 | 191298304 | 6539 | 726 | 586944 | 500 |
| ARFC8 | 89995990 | 13499398500 | 150 | 0.9933 | 0.9785 | 0.4616 | 89265728 | 13347507051 | 149 | 0.9951 | 0.982 | 0.4593 | 80566 | 177948302 | 6239 | 712 | 379617 | 500 |
| ARFC9 | 81079810 | 12161971500 | 150 | 0.9931 | 0.9776 | 0.4642 | 80481562 | 12042048876 | 149 | 0.9947 | 0.9807 | 0.4622 | 107109 | 207057332 | 3706 | 673 | 346422 | 500 |
| HC1 | 94165882 | 14124882300 | 150 | 0.993 | 0.9776 | 0.4934 | 93538696 | 13965664515 | 149 | 0.9948 | 0.981 | 0.4919 | 81670 | 190374832 | 6532 | 732 | 585839 | 500 |
| HC10 | 99128408 | 14869261200 | 150 | 0.9937 | 0.9794 | 0.4354 | 98611406 | 14737426064 | 149 | 0.9951 | 0.9821 | 0.4339 | 107783 | 233236656 | 6112 | 686 | 560844 | 500 |
| HC11 | 83098862 | 12464829300 | 150 | 0.9921 | 0.9747 | 0.4694 | 82445122 | 12314134983 | 149 | 0.9941 | 0.9784 | 0.4672 | 111500 | 204345104 | 3778 | 643 | 496208 | 500 |
| HC12 | 79092224 | 11863833600 | 150 | 0.9912 | 0.9729 | 0.5102 | 77559682 | 11611676309 | 149 | 0.9942 | 0.979 | 0.5048 | 94424 | 194353031 | 4115 | 708 | 425669 | 500 |
| HC13 | 90520486 | 13578072900 | 150 | 0.993 | 0.9779 | 0.4701 | 89716932 | 13393392281 | 149 | 0.995 | 0.9818 | 0.4676 | 127972 | 265116523 | 5426 | 671 | 549483 | 500 |
| HC14 | 80083220 | 12012483000 | 150 | 0.9935 | 0.9788 | 0.4585 | 79552234 | 11907104756 | 149 | 0.9949 | 0.9816 | 0.4567 | 130310 | 261229289 | 4368 | 678 | 483033 | 500 |
| HC15 | 106052512 | 15907876800 | 150 | 0.9929 | 0.9775 | 0.4833 | 105205034 | 15743663925 | 149 | 0.9947 | 0.9809 | 0.4816 | 94842 | 215964668 | 5542 | 734 | 501174 | 500 |
| HC16 | 69437090 | 10415563500 | 150 | 0.9932 | 0.9779 | 0.5018 | 69006600 | 10325421827 | 149 | 0.9947 | 0.9808 | 0.5005 | 120236 | 238989924 | 4305 | 681 | 762592 | 500 |
| HC17 | 90098730 | 13514809500 | 150 | 0.9929 | 0.9774 | 0.4885 | 89455572 | 13345817302 | 149 | 0.9948 | 0.981 | 0.4866 | 128855 | 284298342 | 6102 | 709 | 424251 | 500 |
| HC2 | 83448354 | 12517253100 | 150 | 0.9903 | 0.9726 | 0.4704 | 81483854 | 12177470663 | 149 | 0.9947 | 0.981 | 0.4643 | 68687 | 167466844 | 6726 | 737 | 443409 | 500 |
| HC3 | 91055814 | 13658372100 | 150 | 0.9933 | 0.9779 | 0.5114 | 90610170 | 13566706444 | 149 | 0.9946 | 0.9804 | 0.5106 | 88516 | 151308809 | 3324 | 618 | 318822 | 500 |
| HC4 | 72586598 | 10887989700 | 150 | 0.9931 | 0.9776 | 0.5045 | 72218180 | 10795946086 | 149 | 0.9945 | 0.9803 | 0.5035 | 68953 | 145787211 | 5570 | 687 | 470880 | 500 |
| HC5 | 83079034 | 12461855100 | 150 | 0.9932 | 0.9773 | 0.5099 | 82666224 | 12379690480 | 149 | 0.9945 | 0.9797 | 0.5092 | 127626 | 263452971 | 4407 | 697 | 595844 | 500 |
| HC6 | 62908958 | 9436343700 | 150 | 0.9932 | 0.9779 | 0.4944 | 62547306 | 9339558102 | 149 | 0.9948 | 0.981 | 0.4931 | 137664 | 229801679 | 2558 | 637 | 409350 | 500 |
| HC7 | 81610566 | 12241584900 | 150 | 0.9926 | 0.9762 | 0.5049 | 80966538 | 12094954861 | 149 | 0.9944 | 0.9798 | 0.5028 | 99929 | 196279147 | 4411 | 670 | 421506 | 500 |
| HC8 | 77497028 | 11624554200 | 150 | 0.9906 | 0.9722 | 0.4884 | 75356524 | 11241835436 | 149 | 0.9948 | 0.9812 | 0.4793 | 71214 | 150537341 | 7027 | 676 | 465601 | 500 |
| HC9 | 99390898 | 14908634700 | 150 | 0.9926 | 0.9765 | 0.4726 | 98390298 | 14679480138 | 149 | 0.9948 | 0.9809 | 0.4696 | 115539 | 257147330 | 5654 | 733 | 424868 | 500 |
