# Supplementary material for: Gut mycobiota dysbiosis and an emergent state of “co-dysbiosis” are associated with IgE sensitization in children with comorbid allergic rhinitis and constipation
Source: Front Immunol. 2026 Jan 23;16:1745580. doi: 10.3389/fimmu.2025.1745580 (PMC12876214; doi:10.3389/fimmu.2025.1745580)
Supplement: Supplementary file 2 [file Table2.docx]

| Name | ARF-Mean(%) | ARF-Sd(%) | HC-Mean(%) | HC-Sd(%) | Pvalue | Corrected pvalue | Lower ci | Upper ci | Effectsize |
| --- | --- | --- | --- | --- | --- | --- | --- | --- | --- |
| f__Saccharomycetaceae | 20.82929 | 32.28313 | 20.7853 | 27.37123 | 0.5234 | 0.5616 | -18.39 | 19.95 | 0.04417 |
| f__Ambisporaceae | 15.15521 | 31.30906 | 12.72781 | 26.82365 | 0.902 | 0.9237 | -17.6 | 20.84 | 2.428 |
| f__Neocallimastigaceae | 17.06273 | 33.26017 | 9.8622 | 25.74753 | 0.8406 | 0.8821 | -11.93 | 25.16 | 7.201 |
| f__Tuberaceae | 13.69839 | 21.79472 | 4.37576 | 12.06745 | 0.2789 | 0.3422 | -1.998 | 20.84 | 9.322 |
| f__Hypocreaceae | 4.60693 | 20.08116 | 5.27101 | 14.55159 | 0.2676 | 0.3422 | -11.37 | 11.61 | -0.6644 |
| f__Kickxellaceae | 5.07168 | 8.57668 | 4.38885 | 8.28193 | 0.4374 | 0.4828 | -4.984 | 6.079 | 0.6831 |
| f__unclassified_p__Microsporidia | 3.05472 | 11.75647 | 5.22882 | 16.31836 | 0.8918 | 0.9237 | -11.79 | 6.165 | -2.174 |
| f__Cryptobasidiaceae | 4.01674 | 17.50856 | 2.00347 | 4.27701 | 0.06574 | 0.3422 | -3.738 | 10.96 | 2.013 |
| f__Tulasnellaceae | 4.59808 | 14.51661 | 1.15393 | 3.74905 | 0.5286 | 0.5616 | -1.849 | 10.16 | 3.444 |
| f__Gigasporaceae | 1.4872 | 5.43217 | 3.85239 | 4.4138 | 0.01449 | 0.3422 | -5.294 | 1.111 | -2.365 |
| f__Cunninghamellaceae | 0 | 0 | 4.89192 | 19.42292 | 0.1279 | 0.3422 | -14.64 | 0 | -4.892 |
| f__Phaeosphaeriaceae | 0.67622 | 2.94755 | 4.13812 | 14.09695 | 0.474 | 0.5166 | -11.16 | 1.353 | -3.462 |
| f__Marasmiaceae | 0 | 0 | 4.79685 | 18.80441 | 0.05624 | 0.3422 | -14.21 | 0 | -4.797 |
| f__Stachybotryaceae | 4.53062 | 19.74852 | 0.01923 | 0.07693 | 0.9672 | 0.9672 | -0.03846 | 13.59 | 4.511 |
| f__Patellariaceae | 1.11219 | 3.41051 | 2.74206 | 5.47737 | 0.1693 | 0.3422 | -4.774 | 1.053 | -1.63 |
| f__Glomeraceae | 2.80841 | 12.24159 | 0.10536 | 0.42142 | 0.9672 | 0.9672 | -0.3161 | 8.425 | 2.703 |
| f__Xylariaceae | 0 | 0 | 2.69635 | 10.00092 | 0.1279 | 0.3422 | -8.089 | 0 | -2.696 |
| f__Hyaloscyphaceae | 0 | 0 | 2.14286 | 8.57144 | 0.3019 | 0.3422 | -6.429 | 0 | -2.143 |
| f__Gloniaceae | 0.2995 | 1.30548 | 1.74413 | 3.26889 | 0.04643 | 0.3422 | -3.12 | -0.03448 | -1.445 |
| f__Trichosporonaceae | 0 | 0 | 1.27363 | 5.09451 | 0.3019 | 0.3422 | -3.821 | 0 | -1.274 |
| f__Hoehnelomycetaceae | 0 | 0 | 1.13834 | 4.55337 | 0.3019 | 0.3422 | -3.414 | 0 | -1.138 |
| f__Letrouitiaceae | 0.99211 | 4.32451 | 0 | 0 | 0.3896 | 0.4358 | 0 | 2.976 | 0.9921 |
