# Supplementary material for: Gut mycobiota dysbiosis and an emergent state of “co-dysbiosis” are associated with IgE sensitization in children with comorbid allergic rhinitis and constipation
Source: Front Immunol. 2026 Jan 23;16:1745580. doi: 10.3389/fimmu.2025.1745580 (PMC12876214; doi:10.3389/fimmu.2025.1745580)
Supplement: Supplementary file 3 [file Table3.docx]

| Name | ARF-Mean(%) | ARF-Sd(%) | HC-Mean(%) | HC-Sd(%) | Pvalue | Corrected pvalue | Lower ci | Upper ci | Effectsize |
| --- | --- | --- | --- | --- | --- | --- | --- | --- | --- |
| f__Saccharomycetaceae | 20.829287 | 32.28313 | 20.785301 | 27.371226 | 0.5234 | 0.5616 | -18.39 | 19.95 | 0.04417 |
| f__Ambisporaceae | 15.155209 | 31.309064 | 12.727806 | 26.823651 | 0.902 | 0.9237 | -17.6 | 20.84 | 2.428 |
| f__Neocallimastigaceae | 17.062726 | 33.260173 | 9.8622 | 25.747529 | 0.8406 | 0.8821 | -11.93 | 25.16 | 7.201 |
| f__Tuberaceae | 13.698387 | 21.79472 | 4.37576 | 12.06745 | 0.2789 | 0.3422 | -1.998 | 20.84 | 9.322 |
| f__Hypocreaceae | 4.606935 | 20.081162 | 5.271012 | 14.551592 | 0.2676 | 0.3422 | -11.37 | 11.61 | -0.6644 |
| f__Kickxellaceae | 5.071677 | 8.576679 | 4.388853 | 8.281931 | 0.4374 | 0.4828 | -4.984 | 6.079 | 0.6831 |
| f__unclassified_p__Microsporidia | 3.05472 | 11.756473 | 5.228823 | 16.318363 | 0.8918 | 0.9237 | -11.79 | 6.165 | -2.174 |
| f__Cryptobasidiaceae | 4.016739 | 17.508559 | 2.003469 | 4.277015 | 0.06574 | 0.3422 | -3.738 | 10.96 | 2.013 |
| f__Tulasnellaceae | 4.59808 | 14.51661 | 1.15393 | 3.749051 | 0.5286 | 0.5616 | -1.849 | 10.16 | 3.444 |
| f__Gigasporaceae | 1.487197 | 5.432166 | 3.852388 | 4.4138 | 0.01449 | 0.3422 | -5.294 | 1.111 | -2.365 |
| f__Cunninghamellaceae | 0 | 0 | 4.891921 | 19.422921 | 0.1279 | 0.3422 | -14.64 | 0 | -4.892 |
| f__Phaeosphaeriaceae | 0.676215 | 2.947554 | 4.138124 | 14.096947 | 0.474 | 0.5166 | -11.16 | 1.353 | -3.462 |
| f__Marasmiaceae | 0 | 0 | 4.796847 | 18.804415 | 0.05624 | 0.3422 | -14.21 | 0 | -4.797 |
| f__Stachybotryaceae | 4.530621 | 19.748518 | 0.019232 | 0.076927 | 0.9672 | 0.9672 | -0.03846 | 13.59 | 4.511 |
| f__Patellariaceae | 1.112186 | 3.410512 | 2.742059 | 5.477365 | 0.1693 | 0.3422 | -4.774 | 1.053 | -1.63 |
| f__Glomeraceae | 2.808413 | 12.241588 | 0.105355 | 0.421422 | 0.9672 | 0.9672 | -0.3161 | 8.425 | 2.703 |
| f__Xylariaceae | 0 | 0 | 2.696348 | 10.000915 | 0.1279 | 0.3422 | -8.089 | 0 | -2.696 |
| f__Hyaloscyphaceae | 0 | 0 | 2.14286 | 8.57144 | 0.3019 | 0.3422 | -6.429 | 0 | -2.143 |
| f__Gloniaceae | 0.299498 | 1.305483 | 1.744132 | 3.268891 | 0.04643 | 0.3422 | -3.12 | -0.03448 | -1.445 |
| f__Trichosporonaceae | 0 | 0 | 1.273627 | 5.094508 | 0.3019 | 0.3422 | -3.821 | 0 | -1.274 |
| f__Hoehnelomycetaceae | 0 | 0 | 1.138341 | 4.553365 | 0.3019 | 0.3422 | -3.414 | 0 | -1.138 |
| f__Letrouitiaceae | 0.992111 | 4.324513 | 0 | 0 | 0.3896 | 0.4358 | 0 | 2.976 | 0.9921 |
| f__Pucciniaceae | 0 | 0 | 0.662191 | 1.852425 | 0.1279 | 0.3422 | -1.723 | 0 | -0.6622 |
| f__Aspergillaceae | 0 | 0 | 0.475429 | 1.737773 | 0.1279 | 0.3422 | -1.386 | 0 | -0.4754 |
| f__Teratosphaeriaceae | 0 | 0 | 0.403041 | 1.612165 | 0.3019 | 0.3422 | -1.209 | 0 | -0.403 |
| f__Mortierellaceae | 0 | 0 | 0.336435 | 1.345741 | 0.3019 | 0.3422 | -1.009 | 0 | -0.3364 |
| f__Gelatoporiaceae | 0 | 0 | 0.27812 | 1.112479 | 0.3019 | 0.3422 | -0.8343 | 0 | -0.2781 |
| f__Omphalotaceae | 0 | 0 | 0.180263 | 0.721051 | 0.3019 | 0.3422 | -0.5407 | 0 | -0.1802 |
| f__Rhizopodaceae | 0 | 0 | 0.14963 | 0.59852 | 0.3019 | 0.3422 | -0.4489 | 0 | -0.1496 |
| f__Glomerellaceae | 0 | 0 | 0.144415 | 0.57766 | 0.3019 | 0.3422 | -0.4333 | 0 | -0.1444 |
| f__Powellomycetaceae | 0 | 0 | 0.118316 | 0.473264 | 0.3019 | 0.3422 | -0.3549 | 0 | -0.1183 |
| f__Entrophosporaceae | 0 | 0 | 0.09677 | 0.387079 | 0.3019 | 0.3422 | -0.2903 | 0 | -0.09675 |
| f__Mucoraceae | 0 | 0 | 0.094394 | 0.377576 | 0.3019 | 0.3422 | -0.2831 | 0 | -0.09438 |
| f__Basidiobolaceae | 0 | 0 | 0.083706 | 0.334823 | 0.3019 | 0.3422 | -0.2511 | 0 | -0.08369 |
| f__Mycenaceae | 0 | 0 | 0.076662 | 0.306646 | 0.3019 | 0.3422 | -0.2301 | 0 | -0.07669 |
| f__Sclerotiniaceae | 0 | 0 | 0.074559 | 0.298235 | 0.3019 | 0.3422 | -0.2237 | 0 | -0.07456 |
| f__Diversisporaceae | 0 | 0 | 0.065851 | 0.263402 | 0.3019 | 0.3422 | -0.1976 | 0 | -0.06588 |
| f__Spizellomycetaceae | 0 | 0 | 0.063888 | 0.255554 | 0.3019 | 0.3422 | -0.1916 | 0 | -0.06388 |
| f__Umbelopsidaceae | 0 | 0 | 0.0628 | 0.2512 | 0.3019 | 0.3422 | -0.1884 | 0 | -0.06281 |
| f__Pilobolaceae | 0 | 0 | 0.053533 | 0.214133 | 0.3019 | 0.3422 | -0.1606 | 0 | -0.05353 |
| f__Nectriaceae | 0 | 0 | 0.051255 | 0.205021 | 0.3019 | 0.3422 | -0.1538 | 0 | -0.05126 |
| f__Morchellaceae | 0 | 0 | 0.050002 | 0.20001 | 0.3019 | 0.3422 | -0.15 | 0 | -0.05 |
| f__Podoscyphaceae | 0 | 0 | 0.049815 | 0.199259 | 0.3019 | 0.3422 | -0.1494 | 0 | -0.04981 |
| f__Drepanopezizaceae | 0 | 0 | 0.049721 | 0.198886 | 0.3019 | 0.3422 | -0.1492 | 0 | -0.04972 |
| f__Mycosphaerellaceae | 0 | 0 | 0.048306 | 0.193223 | 0.3019 | 0.3422 | -0.1449 | 0 | -0.04831 |
| f__Lichtheimiaceae | 0 | 0 | 0.043635 | 0.174541 | 0.3019 | 0.3422 | -0.1309 | 0 | -0.04364 |
| f__Botryosphaeriaceae | 0 | 0 | 0.041988 | 0.167951 | 0.3019 | 0.3422 | -0.126 | 0 | -0.04199 |
| f__Phycomycetaceae | 0 | 0 | 0.040732 | 0.162927 | 0.3019 | 0.3422 | -0.1222 | 0 | -0.04073 |
| f__Botryobasidiaceae | 0 | 0 | 0.039852 | 0.159407 | 0.3019 | 0.3422 | -0.1196 | 0 | -0.03985 |
| f__Serendipitaceae | 0 | 0 | 0.038087 | 0.152347 | 0.3019 | 0.3422 | -0.1143 | 0 | -0.03809 |
| f__Dimargaritaceae | 0 | 0 | 0.035832 | 0.143327 | 0.3019 | 0.3422 | -0.1075 | 0 | -0.03583 |
| f__Nosematidae | 0 | 0 | 0.034785 | 0.139142 | 0.3019 | 0.3422 | -0.1044 | 0 | -0.03479 |
| f__Polyporaceae | 0 | 0 | 0.032942 | 0.131768 | 0.3019 | 0.3422 | -0.09883 | 0 | -0.03294 |
| f__unclassified_o__Endogonales | 0 | 0 | 0.032592 | 0.130366 | 0.3019 | 0.3422 | -0.09778 | 0 | -0.03259 |
| f__Radiomycetaceae | 0 | 0 | 0.029177 | 0.116709 | 0.3019 | 0.3422 | -0.08753 | 0 | -0.02918 |
| f__Piptocephalidaceae | 0 | 0 | 0.028499 | 0.113995 | 0.3019 | 0.3422 | -0.0855 | 0 | -0.0285 |
| f__Paraglomeraceae | 0 | 0 | 0.028367 | 0.113467 | 0.3019 | 0.3422 | -0.08511 | 0 | -0.02837 |
| f__Myxotrichaceae | 0 | 0 | 0.028199 | 0.112796 | 0.3019 | 0.3422 | -0.0846 | 0 | -0.0282 |
| f__Chytridiaceae | 0 | 0 | 0.027691 | 0.110765 | 0.3019 | 0.3422 | -0.08308 | 0 | -0.02769 |
| f__Irpicaceae | 0 | 0 | 0.0276 | 0.1104 | 0.3019 | 0.3422 | -0.0828 | 0 | -0.0276 |
| f__Lyophyllaceae | 0 | 0 | 0.026185 | 0.104739 | 0.3019 | 0.3422 | -0.07856 | 0 | -0.02619 |
| f__Physodermataceae | 0 | 0 | 0.025215 | 0.10086 | 0.3019 | 0.3422 | -0.07564 | 0 | -0.02521 |
| f__Cordycipitaceae | 0 | 0 | 0.025099 | 0.100395 | 0.3019 | 0.3422 | -0.0753 | 0 | -0.0251 |
| f__unclassified_p__Ascomycota | 0 | 0 | 0.024692 | 0.098769 | 0.3019 | 0.3422 | -0.07408 | 0 | -0.02469 |
| f__Debaryomycetaceae | 0 | 0 | 0.02446 | 0.09784 | 0.3019 | 0.3422 | -0.07339 | 0 | -0.02446 |
| f__Erysiphaceae | 0 | 0 | 0.024314 | 0.097257 | 0.3019 | 0.3422 | -0.07294 | 0 | -0.02431 |
| f__Sigmoideomycetaceae | 0 | 0 | 0.023888 | 0.095551 | 0.3019 | 0.3422 | -0.07166 | 0 | -0.02389 |
| f__Ancylistaceae | 0 | 0 | 0.023888 | 0.095551 | 0.3019 | 0.3422 | -0.07166 | 0 | -0.02389 |
| f__Podosporaceae | 0 | 0 | 0.023578 | 0.09431 | 0.3019 | 0.3422 | -0.07073 | 0 | -0.02358 |
| f__Atheliaceae | 0 | 0 | 0.023 | 0.092 | 0.3019 | 0.3422 | -0.069 | 0 | -0.023 |
| f__unclassified_o__Tremellales | 0 | 0 | 0.022693 | 0.090774 | 0.3019 | 0.3422 | -0.06808 | 0 | -0.02269 |
| f__Plectosphaerellaceae | 0 | 0 | 0.020024 | 0.080094 | 0.3019 | 0.3422 | -0.06008 | 0 | -0.02003 |
| f__Endogonaceae | 0 | 0 | 0.019877 | 0.07951 | 0.3019 | 0.3422 | -0.05963 | 0 | -0.01988 |
| f__Sporidiobolaceae | 0 | 0 | 0.019124 | 0.076495 | 0.3019 | 0.3422 | -0.05738 | 0 | -0.01913 |
| f__Helotiaceae | 0 | 0 | 0.018911 | 0.075645 | 0.3019 | 0.3422 | -0.05674 | 0 | -0.01891 |
| f__Enterocytozoonidae | 0 | 0 | 0.018155 | 0.072619 | 0.3019 | 0.3422 | -0.05447 | 0 | -0.01816 |
| f__Crepidotaceae | 0 | 0 | 0.017637 | 0.070549 | 0.3019 | 0.3422 | -0.05291 | 0 | -0.01764 |
| f__Herpotrichiellaceae | 0 | 0 | 0.017345 | 0.069381 | 0.3019 | 0.3422 | -0.05203 | 0 | -0.01734 |
| f__Microbotryaceae | 0 | 0 | 0.015833 | 0.06333 | 0.3019 | 0.3422 | -0.04749 | 0 | -0.01583 |
| f__Quaeritorhizaceae | 0 | 0 | 0.014641 | 0.058564 | 0.3019 | 0.3422 | -0.04393 | 0 | -0.01464 |
| f__Saccotheciaceae | 0 | 0 | 0.013754 | 0.055014 | 0.3019 | 0.3422 | -0.04127 | 0 | -0.01376 |
| f__Agaricaceae | 0 | 0 | 0.012454 | 0.049815 | 0.3019 | 0.3422 | -0.03737 | 0 | -0.01246 |
| f__Trichocomaceae | 0 | 0 | 0.011161 | 0.044643 | 0.3019 | 0.3422 | -0.03349 | 0 | -0.01116 |
| f__Ceratobasidiaceae | 0 | 0 | 0.009326 | 0.037304 | 0.3019 | 0.3422 | -0.02798 | 0 | -0.009325 |
| f__Stictidaceae | 0 | 0 | 0.007281 | 0.029125 | 0.3019 | 0.3422 | -0.02184 | 0 | -0.007281 |
