# Supplementary material for: Gut mycobiota dysbiosis and an emergent state of “co-dysbiosis” are associated with IgE sensitization in children with comorbid allergic rhinitis and constipation
Source: Front Immunol. 2026 Jan 23;16:1745580. doi: 10.3389/fimmu.2025.1745580 (PMC12876214; doi:10.3389/fimmu.2025.1745580)
Supplement: Supplementary file 4 [file Table4.docx]

| Name | ARF-Mean(%) | ARF-Sd(%) | HC-Mean(%) | HC-Sd(%) | Pvalue | Corrected pvalue | Lower ci | Upper ci | Effectsize |
| --- | --- | --- | --- | --- | --- | --- | --- | --- | --- |
| s__Saccharomyces_cerevisiae | 20.535549 | 32.214812 | 19.358966 | 26.463425 | 0.5693 | 0.5912 | -18.6 | 18.59 | 1.176 |
| s__Ambispora_gerdemannii | 15.155209 | 31.309064 | 12.727806 | 26.823651 | 0.902 | 0.9225 | -16.5 | 23.65 | 2.428 |
| s__Piromyces_sp._E2 | 12.482591 | 29.488259 | 5.688348 | 22.75339 | 0.2532 | 0.3314 | -10.2 | 23.54 | 6.794 |
| s__Tuber_indicum | 13.698387 | 21.79472 | 4.347972 | 12.076149 | 0.2789 | 0.3314 | -0.9935 | 20.59 | 9.35 |
| s__Mycoemilia_scoparia | 5.071677 | 8.576679 | 4.328087 | 8.310511 | 0.459 | 0.4918 | -5.099 | 6.121 | 0.7439 |
| s__Anaeromyces_robustus | 4.580135 | 18.904445 | 3.753929 | 11.531748 | 0.5495 | 0.5751 | -7.59 | 12.14 | 0.8263 |
| s__Enteropsectra_breve | 3.05472 | 11.756473 | 5.228823 | 16.318363 | 0.8918 | 0.919 | -12.71 | 8.21 | -2.174 |
| s__Acaromyces_ingoldii | 4.016739 | 17.508559 | 2.003469 | 4.277015 | 0.06574 | 0.3314 | -3.434 | 11.08 | 2.013 |
| s__Tulasnella_sp._JGI-2019a | 4.59808 | 14.51661 | 1.15393 | 3.749051 | 0.5286 | 0.5575 | -1.748 | 10.94 | 3.444 |
| s__Trichoderma_aureoviride | 0 | 0 | 5.271012 | 14.551592 | 0.05624 | 0.3314 | -12.28 | 0 | -5.271 |
| s__Dentiscutata_erythropus | 1.487197 | 5.432166 | 3.699966 | 4.234766 | 0.01449 | 0.3314 | -4.921 | 1.4 | -2.213 |
| s__Cunninghamella_echinulata | 0 | 0 | 4.857881 | 19.431523 | 0.3019 | 0.3314 | -14.57 | 0 | -4.858 |
| s__Paraphoma_chrysanthemicola | 0.676215 | 2.947554 | 4.138124 | 14.096947 | 0.474 | 0.5039 | -11.16 | 1.353 | -3.462 |
| s__Tetrapyrgos_nigripes | 0 | 0 | 4.764619 | 18.812681 | 0.1279 | 0.3314 | -14.24 | 0 | -4.765 |
| s__Trichoderma_reesei | 4.606935 | 20.081162 | 0 | 0 | 0.3896 | 0.4208 | 0 | 13.82 | 4.607 |
| s__Paramyrothecium_foliicola | 4.530621 | 19.748518 | 0.019232 | 0.076927 | 0.9672 | 0.9672 | -0.03846 | 13.59 | 4.511 |
| s__Patellaria_atrata | 1.112186 | 3.410512 | 2.742059 | 5.477365 | 0.1693 | 0.3314 | -4.642 | 1.171 | -1.63 |
| s__Rhizophagus_irregularis | 2.808413 | 12.241588 | 0.082535 | 0.330141 | 0.9672 | 0.9672 | -0.2477 | 8.425 | 2.726 |
| s__Xylaria_telfairii | 0 | 0 | 2.505692 | 10.022767 | 0.3019 | 0.3314 | -7.517 | 0 | -2.506 |
| s__Hyaloscypha_variabilis | 0 | 0 | 2.14286 | 8.57144 | 0.3019 | 0.3314 | -6.429 | 0 | -2.143 |
| s__Cenococcum_geophilum | 0.299498 | 1.305483 | 1.744132 | 3.268891 | 0.04643 | 0.3314 | -3.286 | 0.01559 | -1.445 |
| s__Trichosporon_asahii | 0 | 0 | 1.273627 | 5.094508 | 0.3019 | 0.3314 | -3.821 | 0 | -1.274 |
| s__Atractiella_rhizophila | 0 | 0 | 1.138341 | 4.553365 | 0.3019 | 0.3314 | -3.414 | 0 | -1.138 |
| s__Letrouitia_transgressa | 0.992111 | 4.324513 | 0 | 0 | 0.3896 | 0.4208 | 0 | 2.976 | 0.9921 |
| s__Saccharomyces_cerevisiae_x_Saccharomyces_kudriavzevii | 0 | 0 | 0.673531 | 1.668461 | 0.004392 | 0.3314 | -1.547 | -0.06398 | -0.6735 |
| s__Saccharomyces_cariocanus | 0 | 0 | 0.620181 | 1.221156 | 0.01056 | 0.3314 | -1.236 | -0.08134 | -0.6202 |
| s__Aspergillus_heteromorphus | 0 | 0 | 0.435269 | 1.741075 | 0.3019 | 0.3314 | -1.306 | 0 | -0.4352 |
| s__Kluyveromyces_marxianus | 0.293739 | 1.280377 | 0.124797 | 0.499189 | 0.9672 | 0.9672 | -0.3744 | 0.8811 | 0.1689 |
| s__Friedmanniomyces_endolithicus | 0 | 0 | 0.403041 | 1.612165 | 0.3019 | 0.3314 | -1.209 | 0 | -0.403 |
| s__Puccinia_coronata | 0 | 0 | 0.357325 | 1.094194 | 0.1279 | 0.3314 | -1.053 | 0 | -0.3573 |
| s__Piromyces_sp. | 0 | 0 | 0.33182 | 1.327281 | 0.3019 | 0.3314 | -0.9955 | 0 | -0.3318 |
| s__Gelatoporia_subvermispora | 0 | 0 | 0.27812 | 1.112479 | 0.3019 | 0.3314 | -0.8343 | 0 | -0.2781 |
| s__Haplosporangium_gracile | 0 | 0 | 0.24485 | 0.979399 | 0.3019 | 0.3314 | -0.7346 | 0 | -0.2449 |
| s__Puccinia_graminis | 0 | 0 | 0.192105 | 0.76842 | 0.3019 | 0.3314 | -0.5764 | 0 | -0.1921 |
| s__Lentinula_aff._detonsa | 0 | 0 | 0.180263 | 0.721051 | 0.3019 | 0.3314 | -0.5407 | 0 | -0.1802 |
| s__Xylaria_curta | 0 | 0 | 0.172741 | 0.690963 | 0.3019 | 0.3314 | -0.5182 | 0 | -0.1727 |
| s__Rhizopus_delemar | 0 | 0 | 0.14963 | 0.59852 | 0.3019 | 0.3314 | -0.4489 | 0 | -0.1496 |
| s__Puccinia_striiformis | 0 | 0 | 0.112761 | 0.451045 | 0.3019 | 0.3314 | -0.3382 | 0 | -0.1127 |
| s__Scutellospora_calospora | 0 | 0 | 0.098572 | 0.394288 | 0.3019 | 0.3314 | -0.2957 | 0 | -0.09856 |
| s__Entrophospora_candida | 0 | 0 | 0.09677 | 0.387079 | 0.3019 | 0.3314 | -0.2902 | 0 | -0.09675 |
| s__Neocallimastix_cameroonii | 0 | 0 | 0.088103 | 0.352414 | 0.3019 | 0.3314 | -0.2644 | 0 | -0.08812 |
| s__Basidiobolus_meristosporus | 0 | 0 | 0.083706 | 0.334823 | 0.3019 | 0.3314 | -0.2511 | 0 | -0.08368 |
| s__Diversispora_epigaea | 0 | 0 | 0.065851 | 0.263402 | 0.3019 | 0.3314 | -0.1976 | 0 | -0.06587 |
| s__Pilobolus_umbonatus | 0 | 0 | 0.053533 | 0.214133 | 0.3019 | 0.3314 | -0.1606 | 0 | -0.05353 |
| s__Morchella_snyderi | 0 | 0 | 0.050002 | 0.20001 | 0.3019 | 0.3314 | -0.15 | 0 | -0.05 |
| s__Abortiporus_biennis | 0 | 0 | 0.049815 | 0.199259 | 0.3019 | 0.3314 | -0.1494 | 0 | -0.04981 |
| s__Drepanopeziza_brunnea | 0 | 0 | 0.049721 | 0.198886 | 0.3019 | 0.3314 | -0.1492 | 0 | -0.04972 |
| s__Macrophomina_phaseolina | 0 | 0 | 0.041988 | 0.167951 | 0.3019 | 0.3314 | -0.126 | 0 | -0.04199 |
| s__Phycomyces_nitens | 0 | 0 | 0.040732 | 0.162927 | 0.3019 | 0.3314 | -0.1222 | 0 | -0.04073 |
| s__Botryobasidium_botryosum | 0 | 0 | 0.039852 | 0.159407 | 0.3019 | 0.3314 | -0.1195 | 0 | -0.03985 |
| s__Gigaspora_rosea | 0 | 0 | 0.039813 | 0.159252 | 0.3019 | 0.3314 | -0.1194 | 0 | -0.03981 |
| s__Mucor_plumbeus | 0 | 0 | 0.038355 | 0.15342 | 0.3019 | 0.3314 | -0.1151 | 0 | -0.03835 |
| s__Serendipita_vermifera | 0 | 0 | 0.038087 | 0.152347 | 0.3019 | 0.3314 | -0.1143 | 0 | -0.03809 |
| s__Geranomyces_michiganensis | 0 | 0 | 0.0368 | 0.1472 | 0.3019 | 0.3314 | -0.1104 | 0 | -0.0368 |
| s__Dispira_simplex | 0 | 0 | 0.035832 | 0.143327 | 0.3019 | 0.3314 | -0.1075 | 0 | -0.03583 |
| s__Gaertneriomyces_sp._JEL0708 | 0 | 0 | 0.035832 | 0.143327 | 0.3019 | 0.3314 | -0.1075 | 0 | -0.03583 |
| s__Umbelopsis_isabellina | 0 | 0 | 0.035093 | 0.140371 | 0.3019 | 0.3314 | -0.1053 | 0 | -0.03509 |
| s__Coemansia_sp._RSA_475 | 0 | 0 | 0.034913 | 0.139652 | 0.3019 | 0.3314 | -0.1047 | 0 | -0.03491 |
| s__Vairimorpha_ceranae | 0 | 0 | 0.034785 | 0.139142 | 0.3019 | 0.3314 | -0.1044 | 0 | -0.03479 |
| s__Hesseltinella_vesiculosa | 0 | 0 | 0.03404 | 0.13616 | 0.3019 | 0.3314 | -0.1021 | 0 | -0.03404 |
| s__Dactylonectria_macrodidyma | 0 | 0 | 0.033799 | 0.135195 | 0.3019 | 0.3314 | -0.1352 | 0 | -0.0338 |
| s__Trametes_polyzona | 0 | 0 | 0.032942 | 0.131768 | 0.3019 | 0.3314 | -0.09883 | 0 | -0.03294 |
| s__Botryotinia_convoluta | 0 | 0 | 0.032766 | 0.131063 | 0.3019 | 0.3314 | -0.0983 | 0 | -0.03277 |
| s__Bifiguratus_adelaidae | 0 | 0 | 0.032592 | 0.130366 | 0.3019 | 0.3314 | -0.09778 | 0 | -0.03259 |
| s__Colletotrichum_siamense | 0 | 0 | 0.032419 | 0.129677 | 0.3019 | 0.3314 | -0.09725 | 0 | -0.03242 |
| s__Moniliophthora_roreri | 0 | 0 | 0.032227 | 0.128909 | 0.3019 | 0.3314 | -0.09667 | 0 | -0.03222 |
| s__Thoreauomyces_humboldtii | 0 | 0 | 0.031747 | 0.126989 | 0.3019 | 0.3314 | -0.09525 | 0 | -0.03175 |
| s__Zasmidium_cellare | 0 | 0 | 0.030484 | 0.121935 | 0.3019 | 0.3314 | -0.09144 | 0 | -0.03048 |
| s__Geranomyces_variabilis | 0 | 0 | 0.030035 | 0.120141 | 0.3019 | 0.3314 | -0.09011 | 0 | -0.03004 |
| s__Benjaminiella_poitrasii | 0 | 0 | 0.0296 | 0.1184 | 0.3019 | 0.3314 | -0.0888 | 0 | -0.0296 |
| s__Radiomyces_spectabilis | 0 | 0 | 0.029177 | 0.116709 | 0.3019 | 0.3314 | -0.08752 | 0 | -0.02917 |
| s__Syncephalis_pseudoplumigaleata | 0 | 0 | 0.028499 | 0.113995 | 0.3019 | 0.3314 | -0.0855 | 0 | -0.0285 |
| s__Entomortierella_parvispora | 0 | 0 | 0.028367 | 0.113467 | 0.3019 | 0.3314 | -0.0851 | 0 | -0.02837 |
| s__Paraglomus_occultum | 0 | 0 | 0.028367 | 0.113467 | 0.3019 | 0.3314 | -0.0851 | 0 | -0.02837 |
| s__Oidiodendron_maius | 0 | 0 | 0.028199 | 0.112796 | 0.3019 | 0.3314 | -0.0846 | 0 | -0.0282 |
| s__Podila_epicladia | 0 | 0 | 0.028033 | 0.112132 | 0.3019 | 0.3314 | -0.08409 | 0 | -0.02803 |
| s__Aspergillus_candidus | 0 | 0 | 0.02793 | 0.111721 | 0.3019 | 0.3314 | -0.08379 | 0 | -0.02793 |
| s__Tuber_magnatum | 0 | 0 | 0.027788 | 0.111151 | 0.3019 | 0.3314 | -0.08336 | 0 | -0.02779 |
| s__Umbelopsis_vinacea | 0 | 0 | 0.027707 | 0.110828 | 0.3019 | 0.3314 | -0.08312 | 0 | -0.02771 |
| s__Trametopsis_cervina | 0 | 0 | 0.0276 | 0.1104 | 0.3019 | 0.3314 | -0.0828 | 0 | -0.0276 |
| s__Colletotrichum_fioriniae | 0 | 0 | 0.026924 | 0.107697 | 0.3019 | 0.3314 | -0.08077 | 0 | -0.02692 |
| s__Mortierella_alpina | 0 | 0 | 0.026568 | 0.106271 | 0.3019 | 0.3314 | -0.0797 | 0 | -0.02657 |
| s__Thamnidium_elegans | 0 | 0 | 0.026439 | 0.105756 | 0.3019 | 0.3314 | -0.07931 | 0 | -0.02644 |
| s__Termitomyces_sp._J132 | 0 | 0 | 0.026185 | 0.104739 | 0.3019 | 0.3314 | -0.07856 | 0 | -0.02619 |
| s__Coemansia_sp._Benny_D115 | 0 | 0 | 0.025853 | 0.103413 | 0.3019 | 0.3314 | -0.07757 | 0 | -0.02586 |
| s__Paraphysoderma_sedebokerense | 0 | 0 | 0.025215 | 0.10086 | 0.3019 | 0.3314 | -0.07563 | 0 | -0.02521 |
| s__Beauveria_sungii | 0 | 0 | 0.025099 | 0.100395 | 0.3019 | 0.3314 | -0.0753 | 0 | -0.0251 |
| s__Sarcosagium_campestre | 0 | 0 | 0.024692 | 0.098769 | 0.3019 | 0.3314 | -0.07408 | 0 | -0.02469 |
| s__Candida_albicans | 0 | 0 | 0.02446 | 0.09784 | 0.3019 | 0.3314 | -0.07338 | 0 | -0.02446 |
| s__Roridomyces_roridus | 0 | 0 | 0.024314 | 0.097257 | 0.3019 | 0.3314 | -0.07293 | 0 | -0.02431 |
| s__Podosphaera_aphanis | 0 | 0 | 0.024314 | 0.097257 | 0.3019 | 0.3314 | -0.07293 | 0 | -0.02431 |
| s__Conidiobolus_coronatus | 0 | 0 | 0.023888 | 0.095551 | 0.3019 | 0.3314 | -0.07166 | 0 | -0.02389 |
| s__Thamnocephalis_sphaerospora | 0 | 0 | 0.023888 | 0.095551 | 0.3019 | 0.3314 | -0.07166 | 0 | -0.02389 |
| s__Podospora_australis | 0 | 0 | 0.023578 | 0.09431 | 0.3019 | 0.3314 | -0.07072 | 0 | -0.02357 |
| s__Fibularhizoctonia_sp._CBS_109695 | 0 | 0 | 0.023 | 0.092 | 0.3019 | 0.3314 | -0.069 | 0 | -0.023 |
| s__Glomus_cerebriforme | 0 | 0 | 0.02282 | 0.091281 | 0.3019 | 0.3314 | -0.06845 | 0 | -0.02282 |
| s__Tremellales_sp._Uapishka_1 | 0 | 0 | 0.022693 | 0.090774 | 0.3019 | 0.3314 | -0.06808 | 0 | -0.02269 |
| s__Sclerotinia_nivalis | 0 | 0 | 0.022479 | 0.089917 | 0.3019 | 0.3314 | -0.06744 | 0 | -0.02248 |
| s__Mycena_vulgaris | 0 | 0 | 0.022248 | 0.088994 | 0.3019 | 0.3314 | -0.06675 | 0 | -0.02225 |
| s__Colletotrichum_tamarilloi | 0 | 0 | 0.021844 | 0.087376 | 0.3019 | 0.3314 | -0.06553 | 0 | -0.02184 |
| s__Colletotrichum_higginsianum | 0 | 0 | 0.021499 | 0.085996 | 0.3019 | 0.3314 | -0.0645 | 0 | -0.0215 |
| s__Colletotrichum_incanum | 0 | 0 | 0.021467 | 0.085867 | 0.3019 | 0.3314 | -0.0644 | 0 | -0.02147 |
| s__Colletotrichum_orchidophilum | 0 | 0 | 0.020262 | 0.081048 | 0.3019 | 0.3314 | -0.06079 | 0 | -0.02026 |
| s__Verticillium_alfalfae | 0 | 0 | 0.020024 | 0.080094 | 0.3019 | 0.3314 | -0.06007 | 0 | -0.02002 |
| s__Jimgerdemannia_flammicorona | 0 | 0 | 0.019877 | 0.07951 | 0.3019 | 0.3314 | -0.05962 | 0 | -0.01987 |
| s__Fimicolochytrium_jonesii | 0 | 0 | 0.019733 | 0.078934 | 0.3019 | 0.3314 | -0.05919 | 0 | -0.01973 |
| s__Botrytis_cinerea | 0 | 0 | 0.019314 | 0.077254 | 0.3019 | 0.3314 | -0.05794 | 0 | -0.01931 |
| s__Sporobolomyces_salmonicolor | 0 | 0 | 0.019124 | 0.076495 | 0.3019 | 0.3314 | -0.05737 | 0 | -0.01912 |
| s__Bisporella_sp._PMI_857 | 0 | 0 | 0.018911 | 0.075645 | 0.3019 | 0.3314 | -0.05674 | 0 | -0.01891 |
| s__Spizellomyces_sp._'palustris' | 0 | 0 | 0.018601 | 0.074405 | 0.3019 | 0.3314 | -0.0558 | 0 | -0.0186 |
| s__Irineochytrium_annulatum | 0 | 0 | 0.018236 | 0.072943 | 0.3019 | 0.3314 | -0.05471 | 0 | -0.01824 |
| s__Enterospora_canceri | 0 | 0 | 0.018155 | 0.072619 | 0.3019 | 0.3314 | -0.05447 | 0 | -0.01816 |
| s__Xylariaceae_sp._FL0594 | 0 | 0 | 0.017916 | 0.071663 | 0.3019 | 0.3314 | -0.05375 | 0 | -0.01792 |
| s__Zymoseptoria_brevis | 0 | 0 | 0.017822 | 0.071288 | 0.3019 | 0.3314 | -0.05347 | 0 | -0.01782 |
| s__Dichotomocladium_elegans | 0 | 0 | 0.017729 | 0.070917 | 0.3019 | 0.3314 | -0.05319 | 0 | -0.01773 |
| s__Crepidotus_variabilis | 0 | 0 | 0.017637 | 0.070549 | 0.3019 | 0.3314 | -0.05291 | 0 | -0.01764 |
| s__Fusarium_acuminatum | 0 | 0 | 0.017456 | 0.069826 | 0.3019 | 0.3314 | -0.05237 | 0 | -0.01746 |
| s__Exophiala_mesophila | 0 | 0 | 0.017345 | 0.069381 | 0.3019 | 0.3314 | -0.05203 | 0 | -0.01734 |
| s__Mycena_citricolor | 0 | 0 | 0.016879 | 0.067518 | 0.3019 | 0.3314 | -0.05064 | 0 | -0.01688 |
| s__Microbotryum_silenes-dioicae | 0 | 0 | 0.015833 | 0.06333 | 0.3019 | 0.3314 | -0.04749 | 0 | -0.01583 |
| s__Lichtheimia_hyalospora | 0 | 0 | 0.015591 | 0.062364 | 0.3019 | 0.3314 | -0.04678 | 0 | -0.01559 |
| s__Quaeritorhiza_haematococci | 0 | 0 | 0.014641 | 0.058564 | 0.3019 | 0.3314 | -0.04393 | 0 | -0.01464 |
| s__Racocetra_persica | 0 | 0 | 0.014037 | 0.056149 | 0.3019 | 0.3314 | -0.04211 | 0 | -0.01404 |
| s__Aureobasidium_melanogenum | 0 | 0 | 0.013754 | 0.055014 | 0.3019 | 0.3314 | -0.04127 | 0 | -0.01376 |
| s__Mycena_leptocephala | 0 | 0 | 0.013219 | 0.052878 | 0.3019 | 0.3314 | -0.03965 | 0 | -0.01322 |
| s__Leucoagaricus_sp._SymC.cos | 0 | 0 | 0.012454 | 0.049815 | 0.3019 | 0.3314 | -0.03737 | 0 | -0.01246 |
| s__Aspergillus_versicolor | 0 | 0 | 0.01223 | 0.04892 | 0.3019 | 0.3314 | -0.03669 | 0 | -0.01223 |
| s__Thermomyces_lanuginosus | 0 | 0 | 0.011161 | 0.044643 | 0.3019 | 0.3314 | -0.03349 | 0 | -0.01116 |
| s__Lichtheimia_corymbifera | 0 | 0 | 0.010315 | 0.041261 | 0.3019 | 0.3314 | -0.03094 | 0 | -0.01031 |
| s__Phlyctochytrium_planicorne | 0 | 0 | 0.009456 | 0.037822 | 0.3019 | 0.3314 | -0.02837 | 0 | -0.009456 |
| s__Gaertneriomyces_semiglobifer | 0 | 0 | 0.009456 | 0.037822 | 0.3019 | 0.3314 | -0.02837 | 0 | -0.009456 |
| s__Rhizoctonia_solani | 0 | 0 | 0.009326 | 0.037304 | 0.3019 | 0.3314 | -0.02797 | 0 | -0.009325 |
| s__Podila_verticillata | 0 | 0 | 0.008618 | 0.034471 | 0.3019 | 0.3314 | -0.02586 | 0 | -0.008618 |
| s__Lachancea_thermotolerans | 0 | 0 | 0.007825 | 0.031301 | 0.3019 | 0.3314 | -0.02347 | 0 | -0.007825 |
| s__Stictis_urceolata | 0 | 0 | 0.007281 | 0.029125 | 0.3019 | 0.3314 | -0.02184 | 0 | -0.007281 |
